# Evolution of vulnerability of communities facing repeated hazards

**DOI:** 10.1371/journal.pone.0182719

**Published:** 2017-09-27

**Authors:** Allison C. Reilly, Seth D. Guikema, Laiyin Zhu, Takeru Igusa

**Affiliations:** 1 Department of Civil and Environmental Engineering, University of Maryland, College Park, Maryland, United States of America; 2 Department of Industrial and Operations Engineering, University of Michigan, Ann Arbor, Michigan, United States of America; 3 Department of Geography, Western Michigan University, Kalamazoo, Michigan, United States of America; 4 Department of Civil Engineering, Johns Hopkins University, Baltimore, Maryland, United States of America; US Army Engineer Research and Development Center, UNITED STATES

## Abstract

The decisions that individuals make when recovering from and adapting to repeated hazards affect a region’s vulnerability in future hazards. As such, community vulnerability is not a static property but rather a dynamic property dependent on behavioral responses to repeated hazards and damage. This paper is the first of its kind to build a framework that addresses the complex interactions between repeated hazards, regional damage, mitigation decisions, and community vulnerability. The framework enables researchers and regional planners to visualize and quantify how a community could evolve over time in response to repeated hazards under various behavioral scenarios. An illustrative example using parcel-level data from Anne Arundel County, Maryland—a county that experiences fairly frequent hurricanes—is presented to illustrate the methodology and to demonstrate how the interplay between individual choices and regional vulnerability is affected by the region’s hurricane experience.

## Introduction

Hazard-prone communities need to make reoccurring decisions on whether to make upgrades to their built and natural environment, and if so, which upgrades to undertake. Their decisions are based on a bevy of factors, including mitigation costs, their beliefs about the reoccurrence and intensity of future hazards, their beliefs about the protection that upgrades offer, the losses covered by insurance and a centralized government, and social norms [1]. While upgrades generally reduce community vulnerability to future hazards, they may also change the characteristics of the hazard itself. For example, coastal protection (e.g., flood walls, wetland restoration) will lower the likelihood of future coastal surge events. This demonstrates how regional vulnerability is a function of the complex interplay between infrastructure, hazard-mitigation behavior, and the hazard itself.

Infrastructure vulnerability research has made significant progress in the past two decades, and has generally focused on the impact that a single hazard event may cause. Cutter et al. [2] say, however, that “these frameworks often fail to capture antecedent social factors that occur at the most local levels.” In this paper, we make progress toward lessening this gap by developing a modeling framework that can capture and quantify the interactions among hazard environment, the behavioral response to risk, and infrastructure. This framework is useful for analyzing the dynamics of community vulnerability as measured by potential to regional damage [3, 4]. Vulnerability is assumed to be a time-varying, dynamic property dependent on individual choices, the evolving building stock, and damage and not a static property (e.g., as is assumed in [5] and [6]). Our framework can simulate the interactions of individuals who experience repeated hazards, their decisions to mitigate, and potential policy interventions. Through simulation, we assess the time-dependent effects of behavioral scenarios on regional vulnerability processes.

We use a broad class of simulation models as the computational platform, and the specific type of model (discrete-event simulation, cellular automaton, or agent-based model) depends on the assumptions made about whether individuals interact and the specific questions being asked. In the simpler form of the framework, individuals respond to only their hazard environment. The decisions they make have no bearing on the decisions made by other individuals. Their actions (e.g., to mitigate) change their physical state and their vulnerability in future hazards. We consider this to be more in the spirit of discrete-event simulation models. In contrast, it is possible that individuals in a region interact and change one another’s perception of risk. For example, [7] shows that when one person mitigates, others in close proximity are more likely to mitigate, even after controlling for other factors. Here, mitigation is an informal risk communication method that changes individual beliefs to their vulnerability to hazards and affects their decisions. This is more in the spirit of cellular automaton and agent-based models. In the former, the region is divided into grids (e.g., parcels) and the state of each cell in next time-frame depends on the current state of the cell and the current state of the adjacent cells. Agent-based models simulate the actions and interactions of agents within a dynamic environment of interest [8]. More specifically, the decisions made by agents within the model could affect the decisions made by other agents.

Regardless of the computational platform, physical hazard models can be integrated to simulate different hazard environments and the damage caused by these hazards. The model is run over a span of many years to simulate the long-term evolution of regional vulnerability. The modeling framework is capable of bringing insight into many questions including: (1) how the intensity and frequency of repeated hazards and the resulting damage affect behavior and decisions to mitigate, (2) how repeated hazards and individual decisions on mitigation together influence the evolution of regional vulnerability over time, and (3) how regional vulnerability can be affected by behavior. For this last point, the framework allows one to compare vulnerability resulting from behavior based on (3a) individual experiences to hazards, (3b) influences from mitigation choices made by other members of the community, and (3c) policy incentives. The framework is intentionally flexible and can be adapted for any natural hazard for which homeowners can proactively mitigate and for a variety of behavioral assumptions.

The framework’s flexibility has another advantage in that it could aid regional-level policy making aimed at reducing vulnerability. The framework does not demand, in any way, accurate parameterization or calibration of regional hazards or behavioral responses—requiring this for behavior is intuitively unrealistic given the deep uncertainty that exists. Also, given conflicting estimates of how climate change will affect the frequency and intensity of some hazards (e.g., see [9, 10, 11], accurate estimates about future threats might be equally unlikely. Rather, the flexible framework allows researchers and decision-makers to test a variety of hazard and behavioral assumptions via a large ensemble of scenarios and identify policies that are qualitatively robust across a wide range of possibilities. A thoughtful perspective on this topic is provided by Lempert [12].

The goal of this paper is to build and discuss the relevance of a coupled hazard-infrastructure-mitigation simulation model and to provide an illustrative example of how the framework could be used. The flexible framework is comprised of interchangeable modules that can be replaced with different physical models, behavioral assumptions, mitigation strategies, agent interactions, policies, etc., to answer relevant and specific questions that planners may have about their region and how regional vulnerability could evolve. The illustrative example is in its infancy; it uses historical hurricane track data and couples this with a simplistic probabilistic behavioral choice model that mimics how vulnerability could have evolved given varying fractions of the population choosing different mitigation strategies. This could answer questions like, “What would have happened if 10% more people who experienced damage during a hurricane mitigated?” If the outcome is significant, it suggests that mitigation should be encouraged. Future iterations could contain more complex, but equally relevant hazard and behavioral modules.

This framework is not intended to predict how the built environment may look at in the future. Such a predictive method would need to incorporate a multitude of additional dynamic factors, including zoning laws, local and national policies, social preferences and norms, socio-economic environment, and regional productivity, and it would require extensive model validation. And, even if these factors are incorporated, deep uncertainty would still exist. Rather, it is intended to show how regional vulnerability may change and provide a platform for assessing these dynamics.

The first contribution of our paper to the literature is on developing a computational framework that is focused on the interplay among mitigation decisions, infrastructure, and the hazard. Additional contributions are in addressing the research questions (1), (2) and (3a-c) above. We use a detailed example to illustrate the use of the framework in addressing these research questions and to gain insights into the aforementioned interplay. In this illustrative example, we track how vulnerability could evolve for the single-family residential buildings in Anne Arundel County, Maryland, which experiences repeated hurricanes. We also show how variations in the model parameters (e.g., the frequency or intensity of storms) can influence regional vulnerability.

The paper is organized as follows. The second section provides a review on damage estimation and vulnerability models for communities experiencing repeated hazards. In this review, a key gap in the literature is identified: there are no quantitative studies of the influences of individual mitigation behavior on community vulnerability and on the converse influence of hazard intensity and regional damage on individual behavior. The third section titled “Methodology” presents a simulation framework that addresses this research gap, as outlined earlier in this introduction. The fourth section presents the Anne Arundel County illustrative example, with an extensive comparison of community vulnerability under different hurricane environments and behavioral and policy scenarios. The final section provides concluding remarks.

## A review of existing literature

This paper draws from the literature in three key areas. The first area is estimation of physical and economic damage resulting from a natural hazard. The second area is tracking the evolution of regional building stocks via two approaches: a top-down approach in which building stocks evolve due to macro-level forces (e.g., changes to the building code), and a bottom-up approach in which homeowners are independent, heterogeneous decision makers who choose whether to mitigate. The third area relates the use of computational models, and more specifically, simulation models, to model complex systems and regional responses to hazards. The remainder of this section discusses the key areas in more depth.

There exist numerous vulnerability models to estimate both regional physical and economic damage to buildings as a result of a natural hazard such as an earthquake or hurricane (e.g., [13, 14, 15]). The general approach is fairly consistent; first, a physical loading model (e.g., a hurricane wind field model or an earthquake ground shaking intensity model) estimates the relevant hazard loading parameters for each building in the spatial domain. Then either a whole-entity or component-driven approach is used to convert hazard loading into probability of building damage. This is described in more detail below. An economic loss function then converts the damage states into economic losses. A stochastic simulation is typically used to generate multiple replications of this stochastic process, either for a given hazard event or over time with a defined stochastic hazard environment.

The whole-entity approach to assessing damage uses fragility curves. These curves represent the probability of each possible post-event building damage state as a function of the hazard loading on that building [6]. This is the method used in FEMA’s HAZUS-MH [16, 17]. The component method estimates the probability of damage for the components of a single building and models the performance of the building based on structural engineering models while considering interactions among different components [13, 18, 19]. This approach is more complicated than the whole-entity approach and requires significantly more information about each building and computational resources. It does, however, yield more accurate estimates for individual buildings.

With some exceptions, studies examining regional responses to hazards generally do not consider how housing inventories evolve over time, either from individual mitigation decisions or other complex factors, such as land use change and societal preferences. Consideration of how building stocks might evolve is critically important to gain insight into how damage patterns might change, and what might be effective strategies for curbing emerging damage patterns. Further, there has been increased focus on regional adaptation to natural hazards [20, 21, 22]; understanding the drivers of mitigation can aid communities in encouraging cost-effective and inclusive adaptation.

A series of models developed by Jain and Davidson [5, 14, 23] consider changing building stocks that result from modifications to building codes and population growth. These models take a top-down perspective in that they focus on factors that influence similar households equally. While these models are useful in isolating the effects of these specific changes, they do not address the effects of household-level decisions to mitigate in response to damage or government (or e.g., insurance) incentives. That is, a bottom-up perspective is necessary to isolate the effects from an organic regional adaptation process.

The bottom-up simulation approach is more individually focused and thus is dependent on the individual choices that homeowners make. Peng et al. [24] and Kesete et al. [25] are recent examples of models that take a bottom-up approach to understand how building stocks might evolve. Both develop utility theory models of individuals’ decision to insure or retrofit houses and focus on the strategic interactions between the insurers and homeowners. These groups are assumed to have full knowledge of the hazard’s potential (i.e., probability density functions) and want to maximize their utility. Furthermore, the homeowners are assumed to have some degree of risk aversion. These models are important for showing regulators how strategic interactions emerge among the many players in the “mitigation game,” what the consequences of the interactions may be, and how they may be effectively ameliorated. However, descriptive and behavioral economists eschew the notion that all individuals strictly follow the axioms of rational choice. First, knowledge, cognitive, and time limitations exist [26, 27]. That is, in our context, homeowners are typically not fully informed about the hazard and its consequences. Even if they were, they still may *not* make the “best” decision because of the time and cognitive resources required to parse out the “best” decision. Much of the knowledge that homeowners possess is developed over time and through experiences [28] or via social learning and norms [29, 30]. That is, their knowledge of the threat evolves over time as they learn, though this concept is not well studied within the hazard literature.

Bottom-up simulation models that explicitly explore decision-making of interacting agents include cellular automaton models (CAs) and agent-based models (ABMs). In CA models, the region is divided into a grid (e.g., parcels) and each cell within the grid (e.g., each parcel) is assigned a state. In the next time step, the state is updated based on the current state and the states of the adjacent cells [31]. This could be useful in the case of repeated hazards in situations where mitigation is spurred via the action of close neighbors. While CAs are common in the hazards literature, they tend to focus more on the spread of hazards (e.g., forest fires and volcanic lava, [32, 33] or land-use change associated with hazards (e.g., [34, 35]) and not a broader view that focuses on individuals and their preparation in the time between hazards.

The other relevant class of models is ABMs. A distinct advantage of ABMs is that the interaction among agents and their environment produce complex emergent phenomena even when relatively simple behavioral rules are used [36]. Additional value is derived from rigorous sensitivity analysis, which shows how small changes to behavioral rules or the hazard environment could lead to macro-level effects. ABMs are commonly used to model water resources planning [37], financial markets [38, 39], and land-use change [40, 41, 42]. The method, however, is relatively new to research related to community response to natural hazards and the focus here tends to be on evacuation. For example, Chen et al. [43] and Chen and Zhan [44] discuss evacuation prior to a hurricane and the potential effects of network congestion.

## Methodology

We develop a flexible framework based on a simulation environment to track the effects of repeated hazards in a region and to isolate the results of the mitigation choices made by area residents or “agents.” The framework is comprised of many interchangeable modules that can be incorporated to answer different questions. For example, different hazard modules could be used to model (a) different hazard types (e.g., earthquake, hurricanes), (b) non-stationary hazards (e.g., changes to hazard frequency and intensity resulting from climate change), (c) historical, synthetic, worst-case, etc. hazards. Similarly, different behavioral and learning models could be explored. These are all highly relevant because differences in outcomes among these modules show how sensitives the results are to behavior, the hazard environment, etc. This could be used in two specific ways: to quantify the range of possible outcomes and to quantify the importance of understanding the specific aspects of the region. For example, outcomes that are similar despite vastly different behavioral assumptions suggest that an accurate assessment of behavior may be less important for quantifying how regional vulnerability may evolve.

A feature of this platform that is highly relevant to our work is that it allows us to use agents who are not restricted to be homogenous, well-informed and rational. We are able to populate our model with heterogeneous agents who may be influenced by, learn from, and adapt to their complex environment. By attributing unique, yet representative mitigation rules to area residents and then by conducting sensitivity analysis of those rules, we do not seek to answer, “*What mitigation choices are optimal*?” (e.g., as in [45]). Rather, we seek to build an in-silico platform that provides insight into how regional vulnerability to repeated hazards is affected by the decisions made by individuals.

**Fig 1:**
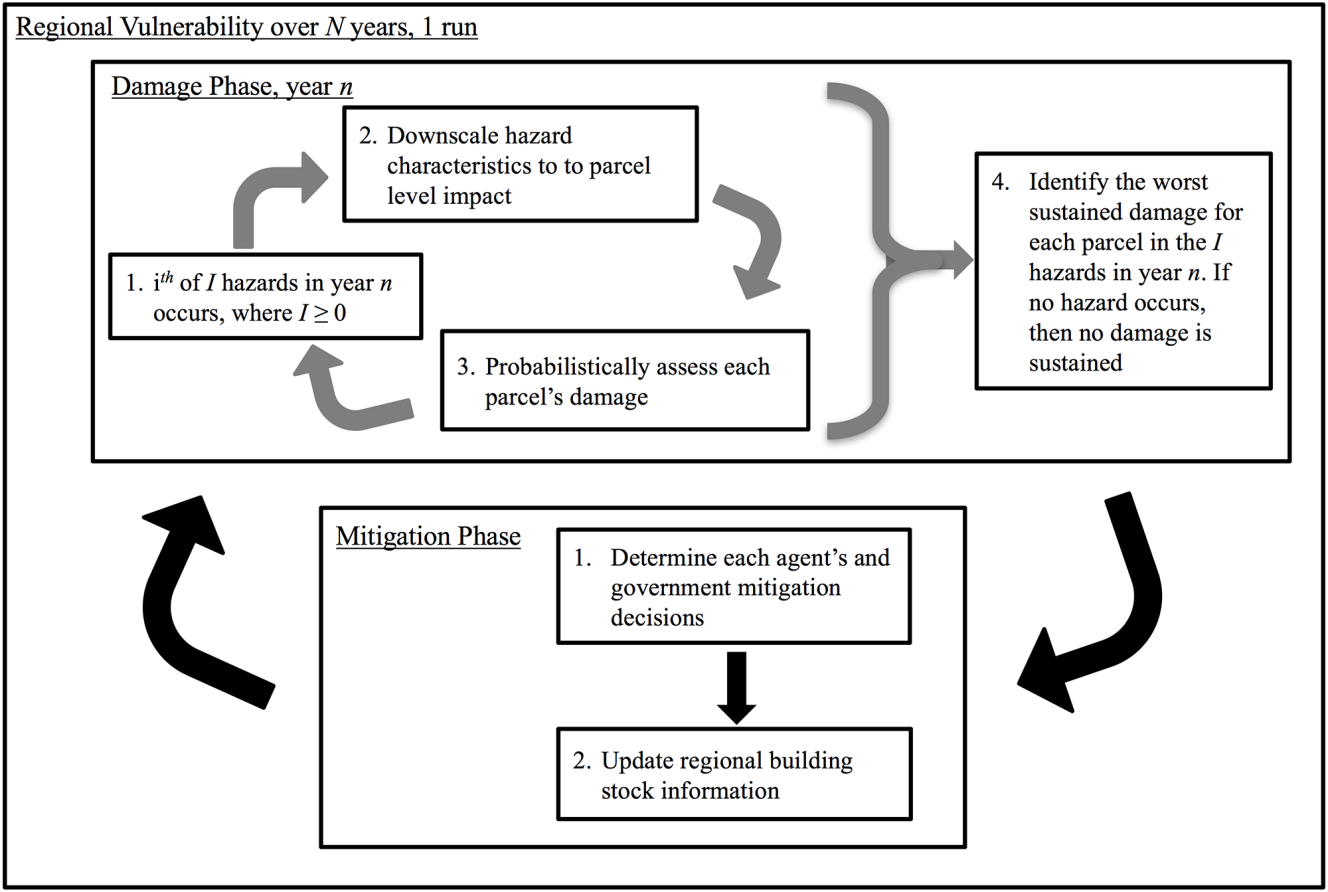
Overview of our framework.

Fig 1 illustrates our unified framework in which different regions, hazard environments, building fragilities, and decision rules are incorporated via computer modules. We initialize the model by choosing a hazard region containing multiple parcels. We implicitly assume that an individual agent owns each parcel and that a centralized government or regional planner exists which may influence the choices of the individual agents and/or make regional changes to the built environment and landscape. Our general framework can be applied to a multitude of hazards, including hurricanes, earthquakes, and tsunamis.

The time-dimension used in the model is discretized into *N* yearly increments. As indicated in Fig 1, the model has two phases for every simulation year: the damage phase and the mitigation phase. During the damage phase, regional hazard events occur and are downscaled to the parcel level. The hazard could result in damage (e.g., structural damage, property damage, electric-power outages, injuries, etc.). In the mitigation phase, agents decide on possible mitigation choices. Many behavioral models are possible for deciding how an agent will choose to mitigate among the possible mitigation choices. This cycle of phases is repeated for each of the *N* years considered in a model run. The value selected for *N* depends on the specific questions being ask by the model. For example, if the model seeks to identify the effectiveness of specific policy interventions (e.g., requiring and enforcement enhanced building codes), a shorter timeframe, such as 20–40 years might be relevant. However, in the present work, we are focused on how communities respond to repeated hurricanes and how different policies may impact this response over time. Hurricanes do not occur frequently in any one location, making a longer time frame for the runs both appropriate and necessary. This study duration also allows for us to answer questions such as “over what timeframe might an intervention start to show benefit.” The illustrative example in the following section uses the region’s entire 162-year hurricane record.

There are multiple sources of stochasticity in an *N*-year model run, so to quantify uncertainties and identify trends, we generate a data set by repeating the model run *M* times. Convergence testing is conducted to ensure that *M* is sufficiently large. The size of the model could be quite large; the illustrative example that follows includes approximately 162,000 agents and has a geographic size of 1523 square kilometers (588 square miles). Details on these simulation steps are given next.

Within each simulation year, zero or more hazards could occur. As mentioned previously, the hazard module that is selected could be one of many, including modules for different hazard types, non-stationary hazard processes, historic hazards, synthetically-generated hazards, etc., and selection depends on the specific questions being asked. Historic hazard data is predefined before the simulation commences and limits the stochasticity of the model. This is acceptable if the questions being ask follows the “what would have happened if…” logic. Synthetically-generated hazards are probabilistically consistent with a region’ experience (or expected future experience), but have not, necessarily, occurred. They can be predefined or generated within the simulation. Many synthetic hazard generation methods (e.g., [46]) can develop a hazard scenario in a very short amount of time. Each hazard’s characteristics are then downscaled to the parcel level so as to measure the intensity of each parcel’s experience and consequences are evaluated. The focus of the illustrative example in the following section is on damage to residential buildings from hurricanes, but other consequences could be assessed too. For example, Reilly et al. [47] assessed the likelihood of electric-power outages due to hurricanes.

The following provides more detail of a damage module that quantifies damage to building stock. First, a building’s resistance level reflects its quality of construction and ability to withstand a particular hazard. The lower the resistance level, the more vulnerable the house is to that hazard. As a building is mitigated and its resistance level increases, the probability of damage given the hazard intensity decreases.

The hazard intensity is coupled with the building characteristics (e.g., building type and resistance level) to probabilistically evaluate damage by using FEMA HAZUS-MH database [16] of building fragility curves. Fragility curves are probabilistic damage exceedance curves and report likely damage levels as a function of hazard intensity, building type, and building quality. Each resistance level has a unique fragility curve. Databases of fragility curves exist for earthquake, flooding, and hurricane (wind) hazards and for numerous types of residential and commercial buildings. These fragility curves are attractive because they are publicly available, have a long history of development and testing [17, 48], and they have been widely used in other hazard damage studies [5, 49].

We acknowledge that HAZUS fragility curves face criticism (e.g., they consider only 36 generic building classes and hence lack resolution) and improvements are possible (e.g., [50, 51]). Our study, however, is not dependent on these particular fragility curves; alternate fragility curves can easily be incorporated into our framework when they become available. Other methods for finding structural and property damage could be relevant too based on the specific objectives (e.g., a component-drive approach [13, 18, 19]).

After a hazard experience, a building may experience no damage or be in damage states one through four. While in reality damage is a continuum, discrete damage states are used for easier representation and binning of damage. Damage state one implies minor house damage, potentially to a window or minor cracks in the façade. A house in damage state two experiences moderate losses, such as toppling of masonry chimneys in an earthquake or extensive damage to the roof cover in a hurricane. Damage state three implies extensive house damage including cracks in the foundations (earthquake) or loss of the roof’s sheathing (hurricane). A house in damage state four experiences complete failure and the structure is typically unsalvageable [16, 47]. We assume that if more than one hazard affects the region, each house’s most severe damage state in year *n* is used. For example, if house *i* experiences two events in year *n*, and its damage state in hazard 1 is three and in hazard 2 is one, then house *i*'s damage state in year *n* is three. Damage is not assumed to compound in subsequent hazards in one year. This is primarily due to limitations with the availability of fragility curves that express compounding damage over subsequent hazards. However, should this type of fragility curved become available, this assumption can be relaxed in future versions of the model.

Once consequences from the hazard are established in year *n*, the mitigation phase begins. The mitigation phase can occur even when no regional damage is sustained. We assume that the mitigation phase begins with agents learning, similar to when individual learns from their experiences. This could affect the choices they make. Agents could update their beliefs over the likelihood of a hazard, the intensity of the hazard, or the damage it might inflict. Learning can be crude, (e.g., an agent reacts to an event but the event has no bearing on future decisions), or be far more sophisticated and follow well-established human learning models (e.g., Bayesian learning and inference [52]). Reilly et al. [53] provides a brief overview of possible learning models.

Next, each agent must choose whether or not to mitigate and by how much based on a set of decision rules and their understanding of the hazard environment. Additionally, it is possible that a regional governing body engages in mitigation on a wide-scale (e.g., subsidize individual mitigation, invest seawalls). More specific to housing damage, a house that is mitigated enters into a higher resistance level. Mitigation can be minor (e.g., adding roof straps or raising mechanicals), major (e.g., making structural changes to the building), or somewhere in between.

A variety of decision models for how agents choose to mitigate can be adopted and ultimately compared. We collapse these decision models into four broad categories: (1) simple, (2) cost-benefit, (3) complex, and (4) and policy. Simple decision rules are typically predefined and easy to implement and loosely mimic the complex decision process that most area residents (e.g., homeowners) undergo. They could be as simple as if-then relationships (e.g., if a house sustains damage, it returns to its original resistance level) or probabilistic (e.g., the likelihood of a house upgrade to a higher resistance level is conditioned on the extent of damage and current resistance level). When combined with rigorous sensitivity analysis, this method can provide measures of macro-level effects from marginal changes in behavior.

Cost-benefit rules assume the costs and benefits can be described via a common unit of measure (e.g., money, utilities) and the agents choose the mitigation alternative that offers the best tradeoff between costs and benefits (e.g., [54]). This can become challenging when trying to assess how agents might value and discount future benefits of mitigation alternatives in highly stochastic hazard environments. For the purposes of cost, it is possible that costs extend beyond the actual cost of mitigation. For example, adding shutters could detract from the aesthetic of the house. Generally speaking, this could be captured monetarily. However, some mitigation efforts could detract from the structural integrity of the house. For example, fortifying the roof increases its structural load. These types of “costs” are captured by the fragility curves.

Complex decision rules for agents are formed using learning and decision models such as Bayesian updating, utility theory, near-miss [55], bounded rationality [56], etc. Depending on risk tolerances, a utility theory model could be construed as a cost-benefit model. Here, each decision is typically stochastic and formed via an agent’s knowledge, which is conditioned on previous experiences and possibly the experience of other agents, combined with its preferences, biases, and willingness to accept risk. An [57] provides an overview of incorporating complex decision modules into ABMs.

It is important to note that not all decision models should be required to conduct a cost-benefit analysis. First, the simple decision rules, like those used in the illustrative example and in many ABMs [e.g., [13, 58], show how certain (reasonably) assumed behaviors may affect outcomes over time. For example, in this context, it could answer a question like “if individuals exhibit some specific characteristic, how long might it take to reach some specific risk-reduction goals?” This could indicate how aggressive decision-makers may need to be to achieve some community-wide objectives. Also, there is evidence that not all individuals rely solely on a cost-benefit analysis when making decisions under risk. For example, prospect theory suggests that biases could induce behaviors that appear otherwise irrational.

Finally, agents could be encouraged or required to mitigate as a result of policy intervention. Here, a community-level policy is issued that either encourages or discourages an agent’s behavior or changes an agent’s knowledge of the hazard. Policies may be simple (e.g., “1% of agents respond to an incentive and mitigate to a higher resistance level”), targeted (e.g., “agents with homes within 1 kilometer of the coastline are eligible for a mitigation subsidy”) [59], or complex (e.g., structural economic models [60]). Further, they could be driven by the behavior or collective action of agents [47, 61]. Subsidies are not the only mechanism to induce mitigation. For example, information campaigns can a cost-effective strategy for inducing mitigation by changing agents’ beliefs about their susceptibility to damage [62]. Using a simple policy model without other decision models can help isolate regional vulnerability reduction when *x*% of agents mitigate. However, it cannot capture the results of complex interactions between regional policies and individual decision-making. The degree to which agents interact dictate whether the model is a discrete-event simulation, a cellular automaton model, or an agent-based model.

Empirical or observational mitigation data could, in theory, be used to parameterize a decision model. Recent survey work has shown why some homeowners choose to reconstruct and mitigate [63]. These studies are helpful to measure the effectiveness of incentive programs tied to mitigation at a regional level and in doing comparative studies across regions experiencing hazards. However, these surveys are spatially and temporally limited and comprehensive damage and mitigation records are generally not available. Spatial and temporal data on damage and mitigation are necessary for a couple of reasons. First, each choice is simply one realization of a complex underlying process that describes an agent’s decision-making process and this process can evolve over time. Temporal data could shed light on this process. Second, Peacock et al. [64] found mitigation decisions are typically tied to perception and understanding of the hazard and past experiences with damage. This suggests that mitigation patterns are location dependent and empirical data from one region should not be ascribed to another. Hence, it was felt that existing historic damage and mitigation records would not provide the information needed to parameterize the decision portion of our model. Should spatial and temporal data become available, we would explore the use of such data in more detail.

Finally, it is possible that, depending on the questions being asked, the behavioral models need not be empirically parameterized. Parameterization could be, for example, aspirational. Researchers might want to know the possible effects of strong social norms on mitigation and regional vulnerability to repeated hazards. Should they discover that strong social norms offer little benefit compared to some other behavioral trait (e.g., recency biases) it suggests (1) policies to incentivize mitigation should not try to enhance social norms and (2) a starting place for where social scientists should conduct more investigation, which, in this case, would be biases and see, if, and how, they are present.

## Illustrative example

### Overview and scope

Our illustrative example focuses on Anne Arundel County, Maryland, and assesses how its regional vulnerability evolves over time in the face of wind damage from repeated hurricanes. More specifically, we seek (1) to isolate how small changes to behavioral decision-making affect long-term regional vulnerability (and thereby seek to keep other factors, like land-use change, constant) and (2) to quantify the time durations that might be necessary to see certain reductions in community vulnerability while assuming certain behaviors. The study also examines the effects of governmental policies and more frequent and intense storms on community vulnerability.

**Fig 2:**
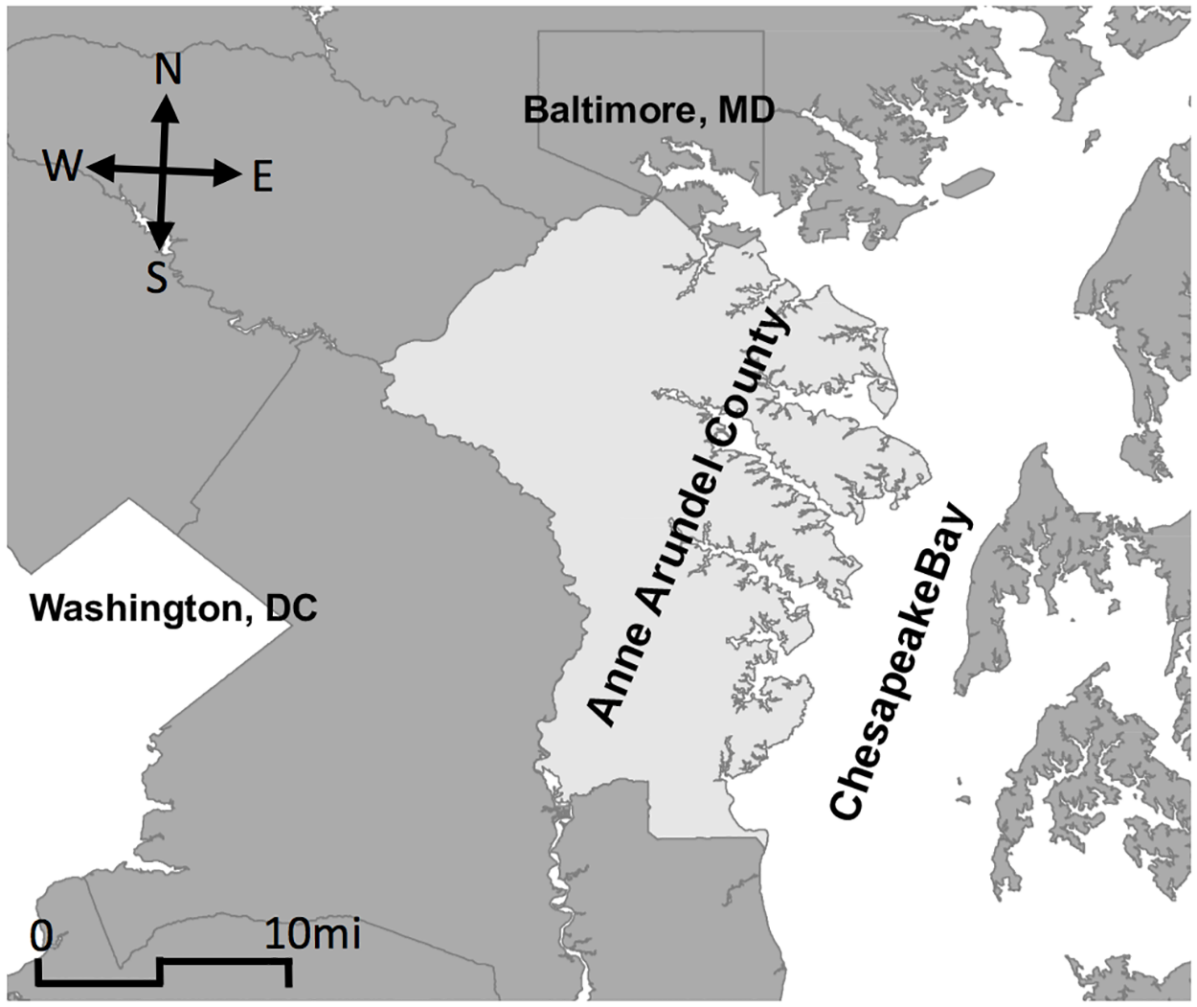
Map of region. [65].

Anne Arundel County is located along the Chesapeake Bay, and between Washington, DC and Baltimore, MD (Fig 2). Its 2010 population is 537,656 [46]. We limit the study to the approximately 162,000 single-family residential houses in Anne Arundel County. As such, the model contains 162,000 agents who are each assumed to own the house in which they reside and have the ability to conduct mitigation. The current illustrative example does not include land use change and thus the number of agents remains constant.

The return period of a hurricane in Anne Arundel County is 12 to 13 years. Protected by the Delmarva Peninsula to its east, its hurricane experience is milder than other regions along the Atlantic Coast.

### Inputs

Housing data are collected from the Maryland Department of Planning [66]. From this data, we classify the County’s single-family building stock into 11 building types defined by the HAZUS-MH based on construction material, dwelling type, and number of stories [12]. More than 96% of the houses are wood-framed homes, with the remainder being a mix of unreinforced masonry, concrete, and mobile homes. The houses’ true resistance levels, i.e., their ability to withstand wind, are unknown. As such, we assume that initially 85% of houses have a resistance level of one, 10% of houses have a resistance level of two, and 5% of houses have a resistance level of three for wood-framed and unreinforced masonry houses and town homes. In addition, 95% of mobile homes and concrete framed houses are assumed to be in resistance level one and 5% in resistance level two. The current building standard for wood-framed houses in study area is resistance level one. Thus, we assume that vast majority of homes are in this resistance level. Resistance levels are randomly assigned to each house at the start of each simulation run. This information is summarized in Tables 1 and 2.

**Table 1:** Description of the housing types, resistance levels, and mitigation strategies for wood-framed and unreinforced masonry houses.

Resistance level	Wood-framed Houses		Unreinforced Masonry Houses	
	(Building Types 1–5)		(Building Types 6–9)	
	Mitigation Strategies	Fraction in Corresponding Resistance Level in Year 1	Mitigation Strategies	Fraction in Corresponding Resistance Level in Year 1
**1**	No mitigation	85%	No mitigation	85%
**2**	Strap	10%	Strap	10%
**3**	Strap + shutters	5%	Strap + improved roof nail spacing	5%
**4**	Strap + improved roof nail spacing	0%	Strap + reinforced masonry	0%
**5**	Strap + improved roof nail spacing + shutters	0%	Strap + improved roof nail spacing + shutters	0%
**6**	Strap + improved roof nail spacing + shutters + hip roof	0%	Strap + improved roof nail spacing + reinforced masonry	0%
**7**	N/A	N/A	Strap + improved roof nail spacing + reinforced masonry + shutters	0%

**Table 2:** Description of the housing types, resistance levels, and mitigation strategies for concrete houses and mobile homes.

Resistance level	Concrete Houses		Mobile Homes	
	(Building Types 10)		(Building Type 11)	
	Mitigation Strategies	Fraction in Corresponding Resistance Level in Year 1	Mitigation Strategies	Fraction in Corresponding Resistance Level in Year 1
**1**	No mitigation	95%	No mitigation	95%
**2**	Shutter	5%	Tie downs	5%
**3**	N/A	N/A	Tie down + shutters	0%

When agents mitigate, they increase the resistance level of their house, which in turn makes them less vulnerable to high winds in future storms. Upgrades could include adding storm shutters, improving roof connections, and adding tie downs for mobile homes. Each building type has a unique set of mitigation options from which agents can choose. For example, agents who own a wood-framed single-story house can change their roof connection, strengthen their roof deck, add storm shutters, or change the shape of their roof. The order of upgrade is dependent on the cost, with less expensive upgrades happening first. Occasionally, two upgrades are approximately the same cost. In the situation where a homeowner chooses to upgrade their house by one resistance level, she will choose either upgrade with a 50% probability. For example, a homeowner with a wood-framed house with roof straps who wishes to upgrade by one resistance level will either install hurricane shutters or change the nail spacing in her roof deck. Tables 1 and 2 and S2:2–S2:5 in the S2 Appendix summarize the mitigation alternatives for each of the building types in order of increasing resistance.

We make some specific but reasonable assumptions for this model. First, agents maintain their homes, and hence houses do not experience natural deterioration or reduction in resistance level due to age. Also, land use change is not explicitly considered; only mitigation is. In future iterations of the model, land use change will be considered when agents make decisions both after hazards and in the years between hazard events. It is important to note that once this occurs, possible home depreciation will be an important feature to capture, especially if depreciation stems from risk perception or other risk considerations. This is especially important if the house depreciates to a level where mitigation becomes no longer cost effective.

Also, we allow only homeowners who have experienced damage to upgrade their house’s construction quality during reconstruction to be more resistant to future hazards. We term this “damage-driven mitigation.” This is to hone in on the impact of previous experience to damage. One hypothesize we could make is that if damage is typically minimal, it could make agents more susceptible to severe damage in intense hazards and the analysis partially seeks to find whether conditions exist that exhibit this.

For simplicity, we use the region’s historical hurricane record since 1851. These data are gathered from the National Hurricane Center’s Historical Tracks Database [67]. In these 162 years, the region experienced 15 hurricanes whose eye was within 120 kilometers of the County’s geographic center and had winds high enough to cause damage. The first storm occurred in the Year 24, the last in Year 162, and the remainder occurred in the time frame between. Years 43 and 105 experienced two storms. Note that using historic tracks is only one option. Alternatively, probabilistically consistent synthetic tracks or worst-case synthetic tracks, among others, may be overlaid on the region. A parametric wind field model [68] is pre-run for each storm to generate each parcel’s peak 3-second sustained wind gust. A typical peak wind gust for any given parcel in an average storm is tropical storm level (i.e., 39 to 73 mph or 17.4m/s to 32.6m/s).

Anne Arundel County’s historical hurricane experience is relatively mild compared to other regions along the eastern US Coast. An interesting comparison is to take the region and to comparatively assess what the impact might be over time under a more severe hurricane experience. For example, one could hypothesize that if damage spurs mitigation, that over a period of some time, that damage rates might be lower after severe storms in environments that experience more powerful storms than damage rates after milder storms in environments that experience less intense storms. To examine how hurricane intensity and frequency influence a community’s vulnerability, we consider the same community and its building stock but subject it to 162 years of hurricane experiences from Miami, Florida. That is, we overlay historical storm tracks affecting Miami on Anne Arundel County and use the same downscaled parametric wind field model to assess parcel-level peak wind gusts. Miami experienced 36 tropical storms or hurricanes in the 162-year record and the average storm intensities are higher than the intensities of Anne Arundel County historical record. If damage sustained from hurricanes spurs mitigation, then houses under the Miami case experience more opportunities to mitigate.

The damage phase uses the historical storm record together with fragility curves from the FEMA HAZUS-MH database [16] to probabilistically assign wind damage. Damage can be none or in one of four damage states. For a given building type, resistance level, and wind speed, the four fragility curves—one for each damage state—provide damage state probabilities. In each replication, each houses’ damage state is randomly chosen in proportion to these probabilities.

### Mitigation scenarios

The mitigation phase considers four mitigation scenarios: (1) the baseline scenario, where damaged houses return to their initial resistance level, (2) the upgrade scenario, where an agent’s decision to mitigate is probabilistic and is a function of the damage it sustains and its current resistance level, (3) the neighbor scenario, which is identical to the upgrade case except houses that are not damaged may be upgraded if neighboring houses are damaged, and (4) the policy case, where the “government” offers yearly mitigation incentives and some fraction of agents take the subsidy and upgrade regardless of whether they experience a storm or damage. The mitigation scenarios are evaluated in separate runs and sensitivity analyses are conducted subsequently to isolate the effects of marginal changes to an individual’s mitigation likelihood.

The baseline scenario is representative of an insurance policy in which homeowners are assumed to possess insurance and receive compensation when their house is damaged. A house that is damaged is repaired to its original resistance level. This assumption is reasonable in many regions of the United States for wind damage. A local insurance agent confirmed that most homeowners in Anne Arundel County (99+%) have homeowners’ insurance and that this pays to restore to either the previous state or to the state corresponding to current building code if damage exceeds 60% of the value of the house [69]. This scenario forms a baseline from which other scenarios are compared.

The upgrade scenario is representative of the fact that some homeowners whose house sustains damage during a storm choose to upgrade their house’s resistance to a level greater than it was before the storm. We implicitly assume that the agents are responsible for the costs associated with the upgrades and costs are not modeled here. The upgrade probabilities reflect two assumptions: (1) that less costly upgrades are more likely to be undertaken when damage is minimal and (2) a house that sustains more damage is more likely to receive a substantial upgrade. Ultimately, the upgrade probabilities are conditioned on each house’s damage state, building type, and resistance level prior to damage. Table 3 shows a subset of the initial upgrade probabilities for the four damage states for single- and two-story wood-framed homes (building types 1 and 2) that are in resistance level 1 prior to damage. These probabilities form a reference against which subsequent, sensitivity analyses are compared. In the sensitivity analyses, the likelihood of an agent mitigating is increased by 10%, 20%, 30%, or 40% or decreased by 10% or 20%. This demonstrates the macro-level effects from these marginal changes in behavior. The remaining transition probabilities are shown in the S2 Appendix. In the illustrative example, any agent who experiences damage either returns to its original resistance level or mitigates because it is assumed that insurance will cover the cost of damage. In future iterations of the framework that consider other hazard and decision models, agents may find it is not cost-effective to return to the previous resistance level after damage due to lack of insurance (e.g., flood insurance is less common to possess) or due to home-value depreciation.

**Table 3:** Upgrade probabilities for one- and two-story wood-framed houses (building types 1–5) that are in resistance level 1 prior to the storm.

Resistance level after mitigation	Damage state after storm			
	1	2	3	4
**1**	60%	40%	5%	0
**2**	39%	50%	0	0
**3**	1%	10%	0	0
**4**	0	0	65%	40%
**5**	0	0	30%	55%
**6**	0	0	0	5%

As an example, a house in resistance level 1 prior to the storm that sustains minimal damage (damage state 1) will upgrade to resistance level 2 by adding roof straps with a 39% likelihood.

The neighbor scenario is nearly identical to the original upgrade scenario; the difference is that parcels near damaged houses may be upgraded as well. This rule represents a scenario where an agent learns from the experience of a neighbor and takes action. Damaged houses use the same upgrade probabilities as in the upgrade scenario. In addition, undamaged houses that have at least some fraction of neighbors within 75 meters who experience at least moderate damage (i.e., at least damage state 2) are upgraded to the next resistance level. This threshold is varied from 10% to 50% to test the effects of mitigation spawned from the experience of different homeowners. In future iterations of this model, this module could be expanded to include a larger social network than neighbors, such as students at the same school.

The fourth scenario is the policy scenario, which represents the possible role of a government providing incentive or subsidies that encourage mitigation. Government subsidies—when applied effectively—can play an important and significant role in reducing community vulnerability [24, 70]. In two separate cases of this scenario, 1% and 3% of agents are assumed to respond to a subsidy and make upgrades to their house. Subsidies are not targeted and all agents are eligible to receive it. Therefore, in the model, 1% or 3% of agents are randomly selected to make an upgrade each year and they upgrade to the next resistance level. This scenario is similar to the baseline scenario except for the yearly subsidy aspect; damaged houses return to their pre-storm resistance level.

The baseline, upgrade, and policy scenario could be viewed as discrete-event simulation, due to the fact that agents do not explicitly learn from nor interact with other agents. The neighbor scenario is more in the spirit of a cellular automaton model, in which each parcel is a cell. An agent’s decision to mitigate affects his or her likelihood of damage in the future, which in turn affects the likelihood that others in close proximity (neighbors) will also mitigate. If the social network were broadened, and agent interaction was considered more explicitly, the model would be more in the spirit of an agent-based model.

Details about the model architecture, computing platform, and run times are provided in the S1 Appendix. A mathematical description is provided in Appendix A. This model is run 500 times for the two hurricane environments (Anne Arundel County and Miami) and for the four mitigation scenarios. With 500 replications, the confidence interval width for the number of damaged houses in the four most severe storms in both environments is less than 0.02% of the mean. Neither building type nor land use patterns change within the model. To reduce confusion with the dates associated with historical building stocks in Anne Arundel County, we label the first year of each simulation as Year 1 –rather than 1851 –with each simulation continuing until Year 162.

### Results

#### Anne Arundel County wind environment

**Fig 3:**
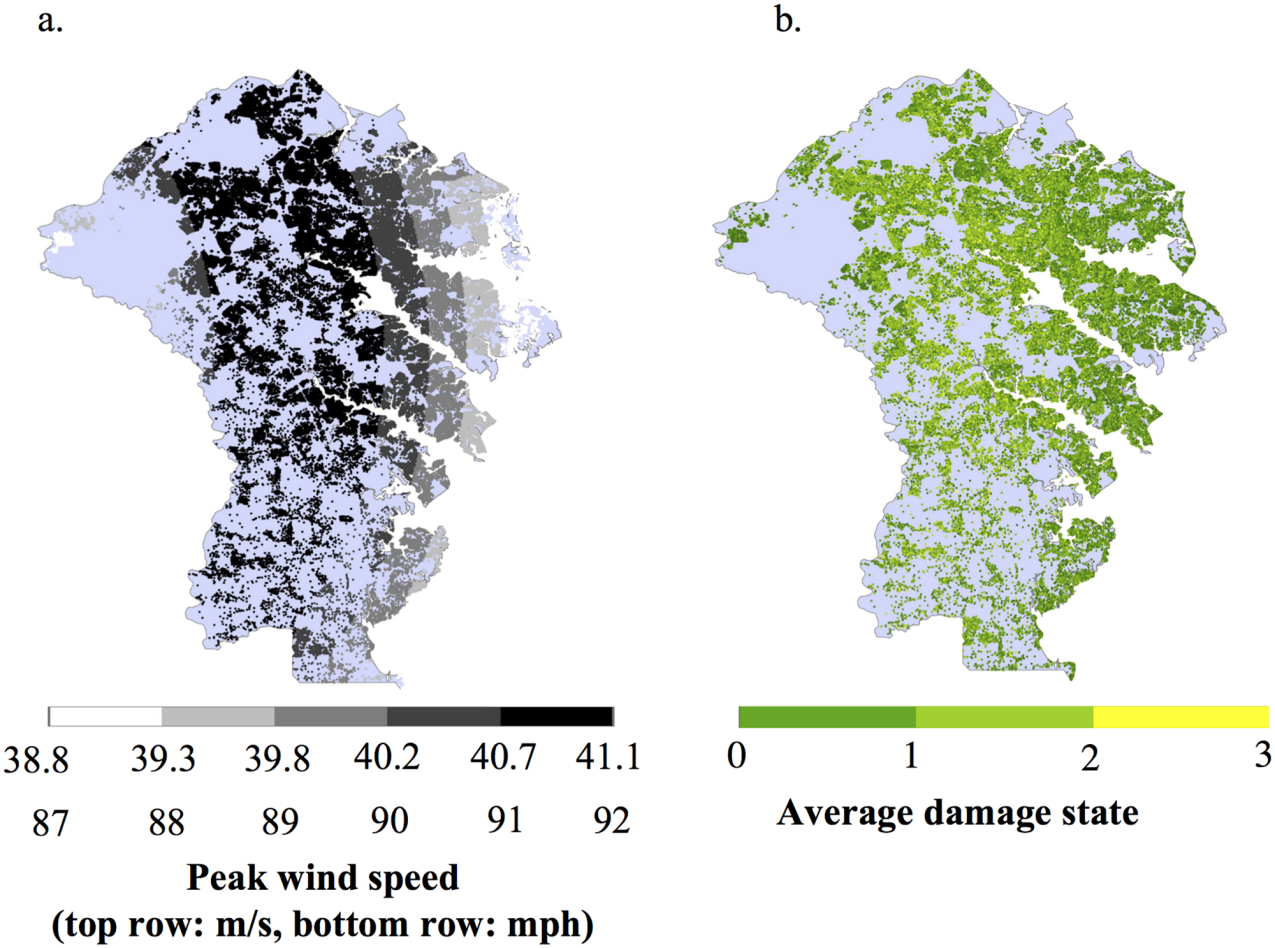
a) Peak wind speed (m/s) for the fourth storm to impact Anne Arundel County, (b) Spatial distribution of damage states, averaged over 500 replications, after the fourth storm for the baseline scenario. **(** The other scenarios are visually similar. Most houses experience no damage or minimal damage. The fourth storm is the region’s most intense storm in its 162-year historical hurricane record.

The three most severe storms in the Anne Arundel County historical record occurred in years 42, 149, and 162. These are the fourth, eleventh, and thirteenth storms, respectively, to affect the County. The fourth storm was the most severe. The regional storm intensities are fairly uniform for all these storms, with the southern region typically experiencing a slightly more intense hazard. As an example, the peak wind speeds for the storm in year 42 are shown in Fig 3a; the maximum wind gust is 41.1 m/s (92 mph), which makes it a Category 1 hurricane at the time of impact for this region. This storm is somewhat uncharacteristic for the region; the storm made landfall in South Carolina and traveled northward through Central Maryland and had relatively minor decay. Hence, inland regions experienced higher wind gusts than coastal regions. The average damage state for each house in the baseline scenario is shown in Fig 3b; the damage is greater where the wind speeds are higher, though the extent of damage is relatively mild.

**Fig 4:**
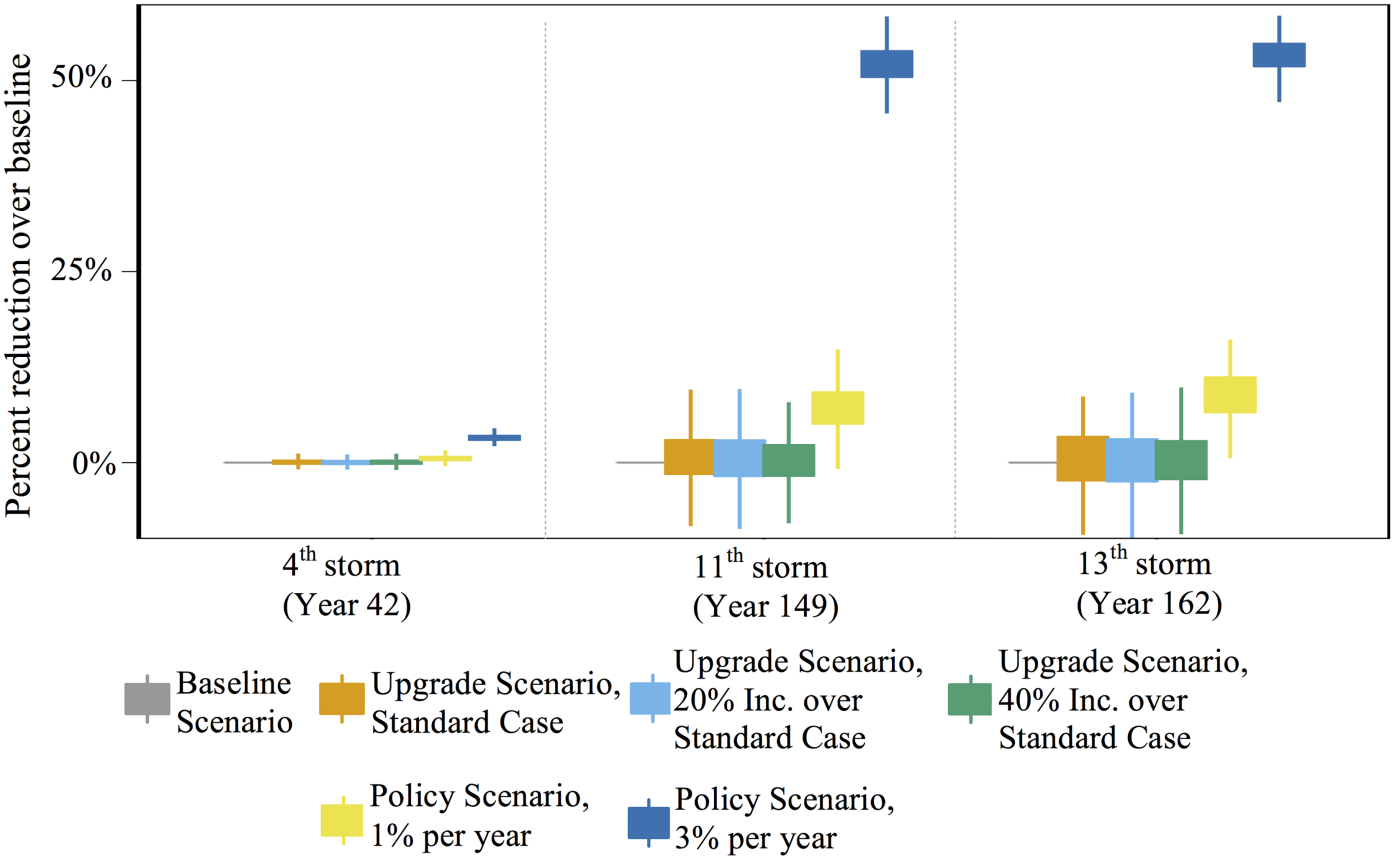
Percent reduction in damage over baseline scenario for the upgrade, and policy scenarios for the three most intense storms in Anne Arundel County’s historical storm record. The region’s most intense storm is its fourth.

Table 4 shows the average number of houses to sustain damage in the three most severe and aforementioned storms in the Anne Arundel County wind environment under the four mitigation scenarios. The results using the reference probabilities (Table 3) are shown for both the upgrade and neighbor scenario and results from sensitivity analyses are presented later. Additionally, the results using a threshold of 10% of neighbors who experience damage are shown for the neighbor case. This table is paired with Fig 4 to show the distribution of damage reduction over the baseline scenario for the same three storms. A positive value means a reduction.

**Table 4:** The average number of houses, averaged over 500 simulations, that sustain damage in the three most severe storms, for the Anne Arundel County hurricane environment, for the baseline, upgrade, neighbor, and policy scenarios.

Case	Damage State				
	All	1	2	3	4
**Baseline**					
Storm 4, Yr 43	57,460	46,014	10,299	276	871
Storm 11, Yr 149	1,490	1,451	39	0	0
Storm 13, Yr 162	1,303	1,271	31	0	1
**Upgrade (Reference)**					
Storm 4, Yr 43	57,440	45,984	10,315	276	865
Storm 11, Yr 149	1,478	1,440	38	0	0
Storm 13, Yr 162	1,298	1,270	28	0	0
**Neighbor (10%)**					
Storm 4, Yr 43	57,436	46,018	10,284	277	857
Storm 11, Yr 149	1,480	1,443	37	0	0
Storm 13, Yr 162	1,287	1,258	29	0	0
**Policy 1%**					
Storm 4, Yr 43	57,148	46,102	10,148	321	577
Storm 11, Yr 149	1,382	1,358	24	0	0
Storm 13, Yr 162	1,185	1,167	18	0	0
**Policy 3%**					
Storm 4, Yr 43	55,586	45,939	9,073	315	259
Storm 11, Yr 149	714	712	2	0	0
Storm 13, Yr 162	606	605	1	0	0

In the baseline scenario, 57,460 houses (or 35% of houses) are expected to experience some damage in the fourth storm and of these damaged houses, 871 (1.5% of damaged houses) are expected to result in total devastation. Far fewer houses are damaged in later storms in this scenario due to the less severe nature of these storms.

Damage drives mitigation in the upgrade and neighbor scenarios. Only 600 houses on average experience hurricane damage prior to the fourth storm (results not shown), and most of that damage is mild. Hence, opportunities to mitigate prior to the fourth storm are minimal and the reduction in damage over the baseline scenario is negligible for these scenarios.

Most houses (98.5%) that are damaged in the fourth storm experience mild to moderate damage (i.e., damage states 1 and 2) and the remainder experience severe damage. In the upgrade and neighbor scenarios, the agents whose houses are severely damaged are more likely to make significant upgrades when they rebuild, though they represent a small fraction of the agents who experience damage and then upgrade in this storm. Most agents that do mitigate make small upgrades—often by adding straps to connect the roof deck to the rest of the house. While this upgrade is inexpensive and often recommended, its benefits are realized only in high winds—speeds that the region does not witness again in the timeframe in the study. Straps reduce the likelihood of extreme damage, not mild damage. Hence, the number of houses that are damaged and number of houses in each damage state, in the 11^th^ and 13^th^ storms, are nearly identical in the baseline, upgrade, and neighbor scenarios and the reduction over baseline is minimal.

The policy cases in this wind environment are more effective at preventing damage simply due to more mitigation opportunities. In the 3% policy scenario, by the fourth storm, 36% of houses (on average) mitigate once, 23% twice, and 10% three times. Replacing toenails with straps is effective at reducing the likelihood of severe damage and the reduced number of houses in damage state 4 reflects this. In fact, there is a 70% reduction of houses in damage state 4 over the baseline scenario in the 3% policy scenario. However, the total reduction in damage is only 3%. This reflects the fact that most of the upgrades chosen thus far only reduce the likelihood of severe damage.

Storms after the fourth also result in less damage under the policy scenarios compared to the other scenarios. For example, after the 13^th^ storm, the 1% policy scenario results in a 7% reduction in overall damage and the 3% scenario results in a 53% reduction over baseline. Unlike before, the primary damage reduction is for houses in damage state 1 and not in damage state 4; the wind speeds in the 13^th^ storm are not high enough to produce severe damage even in the baseline scenario. Hence, it is not the replacement of toenails with straps that drive this reduction in damage because straps do not prevent mild damage. 162 years pass between the start and the 13^th^ storm. In the 1% policy scenario 48% of houses have upgraded at least twice, and 22% at least three times. In the 3% policy scenario, all houses are at their highest resistance level. (This is achieved in approximately 155 years.) Houses in higher resistance levels are more likely to stave off mild damage. Therefore, if a regional objective is to reduce the likelihood of major and minor losses, policymakers should encourage mitigation regardless of the storm experience in that year.

**Fig 5:**
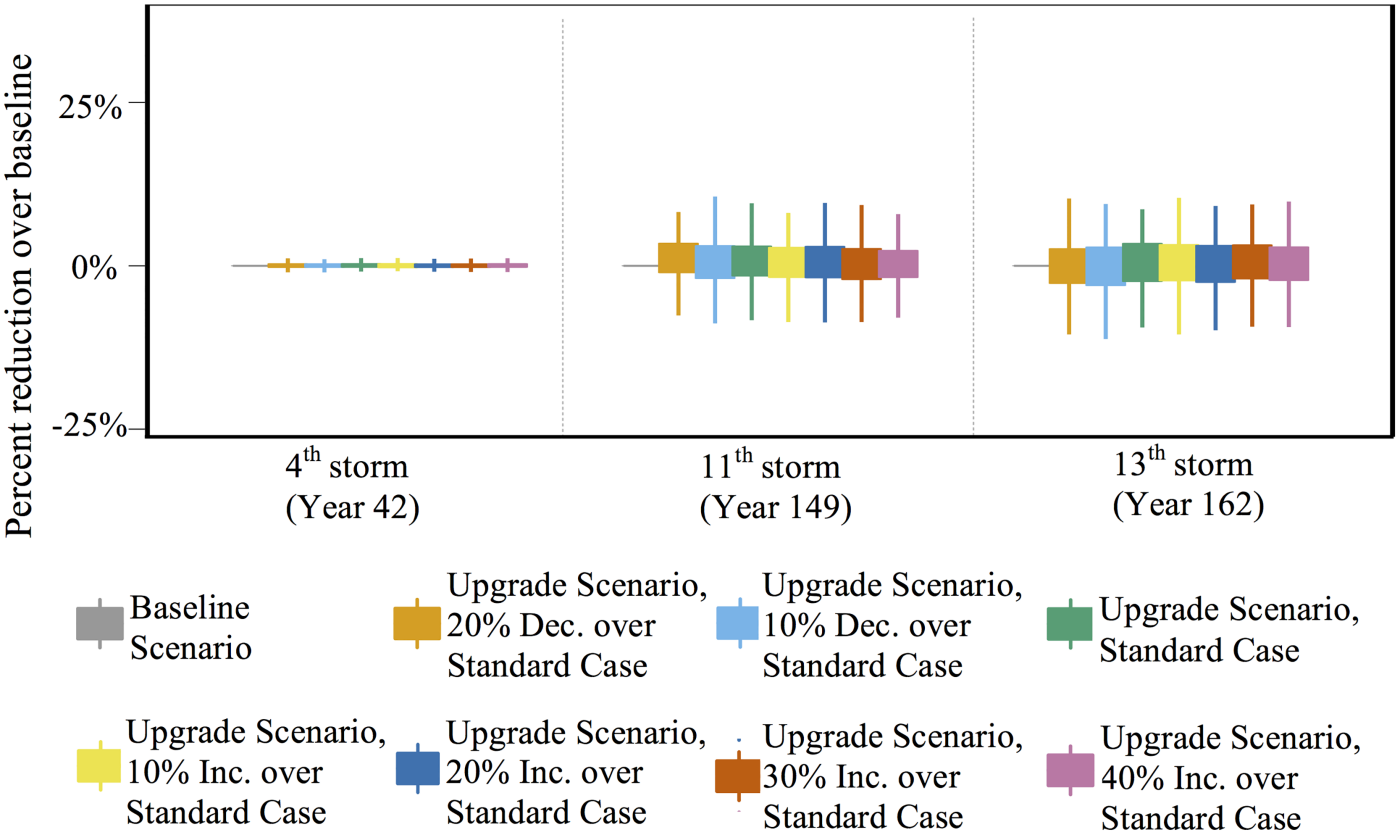
Sensitivity analysis for the upgrade scenario as measured by percent reduction in damage over baseline scenario for the Anne Arundel County’s historical storm record. The transition probabilities are presented in the S2 Appendix and are conditioned on the damage level experienced. The most intense storm is the fourth storm to impact the region.

Sensitivity analyses are conducted for the upgrade scenario (Fig 5). Here, we show how agents are more or less likely to mitigate when they experience damage. We ran the model 500 times for each parameter in the range -20% and 40%, in 10% increments. Because the fourth storm is the only storm to produce substantial damage and the bulk of the mitigation that follows does little to reduce mild damage in future storms, the reduction in damage over baseline is negligible for all storms.

**Fig 6:**
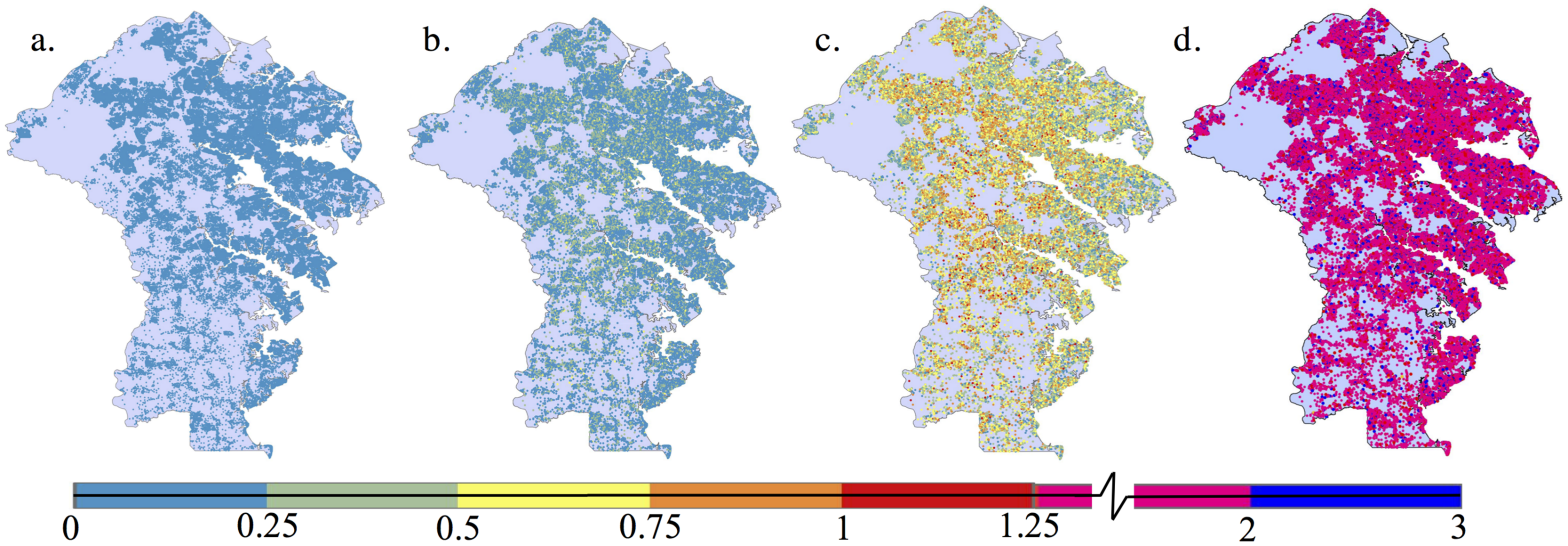
Number of upgrades over 162 years, averaged over 500 replications. (a) Baseline scenario, (b) upgrade scenario (standard case), (c) upgrade scenario (40% greater likelihood of upgrade than the standard case), and (d) 1% policy scenario, for the Anne Arundel County wind environment. All houses make the maximum number of upgrades allowed in the 3% policy scenario. Wood-framed houses may have up to four upgrades, and unreinforced masonry houses may have up to five upgrades. Note how the scale changes after 1.25 upgrades.

Fig 6 shows the spatial distribution of mitigation for the upgrade (standard and 40% over standard) and 1% policy scenarios. Most agents are less than 50% likely to upgrade one resistance level in the standard upgrade scenario (Map 6b). On the other hand, agents are generally between 50% and 100% likely to upgrade by one resistance level in the upgrade scenario that is 40% greater upgrade likelihood than the standard (Map 6c). This upgrade is typically the addition of roof straps. While widespread minimal increase in resistance level does little to thwart mild damage, it offers substantial protection should another strong storm impact the region. In the 1% policy case (Map 6d), most agents increase the resistance level of their house between one and two levels.

In the neighbor scenario, the upgrade decision rule is as follows: If at least *x*% of an undamaged house’s neighbors experience moderate or worse damage during a storm, the undamaged house will upgrade one resistance level. Here, we used several values for *x* between 10% and 50%. The results are approximately the same, regardless of the value used for *x* and there is no reduction in damage over the upgrade case. (For brevity, the results are not plotted.) This is not surprising given relatively mild wind environment. Only (approximately) 10,300 houses ever experience damage state 2. Of these, the damage is typically tightly concentrated, meaning that houses that are damaged are clustered and only a few houses exist within the cluster without any damage.

Of the cases considered, the policy cases in the Anne Arundel County wind environment results in the most reduction in damage and the highest number of upgrades. This is simply due to the hurricane environment not being severe enough to drive mitigation via damage—even when mitigation is probabilistically very likely given any damage. However, it is not intended to imply the policy scenario is cost effective. On the contrary, the policy scenario is a very expensive way to reduce damage in this environment.

The discussion to this point has addressed how small, isolated changes to decision-making following damage could affect regional vulnerability. Another relevant question is, given a particular behavioral response or policy initiative, over what timescale might they be impactful. Returning momentarily to Fig 4, we see that even over very long time scales (i.e., 150 years), the 1% policy scenario is not expected to produce meaningful changes. The 3% policy scenario could produce meaningful changes over very long time scales; however, over time scales more relevant for policy making (i.e., 20–50 years), it is not likely to significantly reduce damage.

#### Miami wind environment

The Miami wind environment is more severe than the Anne Arundel County wind environment both in terms of average wind speed and the number of hurricanes impacting the region. The Miami wind environment is superimposed on Anne Arundel County to test the hypothesis that damage-driven mitigation can be an effective at reducing regional vulnerability and, if true, to what extent. Thirty-six storms affect the region in the 162-year record under consideration. In six storms, at least half of houses experience Category 1 wind speeds, in five storms, at least half of houses experience Category 2 wind speeds, and in four storms, at least half of houses experience Category 3 wind speeds. Table 5 shows the years in which these storms occur.

**Table 5:** The historical hurricane record for the Miami wind environment for storms that affect more than half of houses with Category 1–3 winds.

Category 1		Category 2		Category 3	
Storm Number	Year	Storm Number	Year	Storm Number	Year
13	56	4	20	9	41
21	86	14	59	17	76
25	98	23	95	24	97
29	114	26	100	34	148
35	149	27	102	-	-
36	115	-	-	-	-

Thirty-six storms affect the region with 15 of them being Category 1 or higher. The storm number is the n^th^ storm to affect the region.

**Fig 7:**
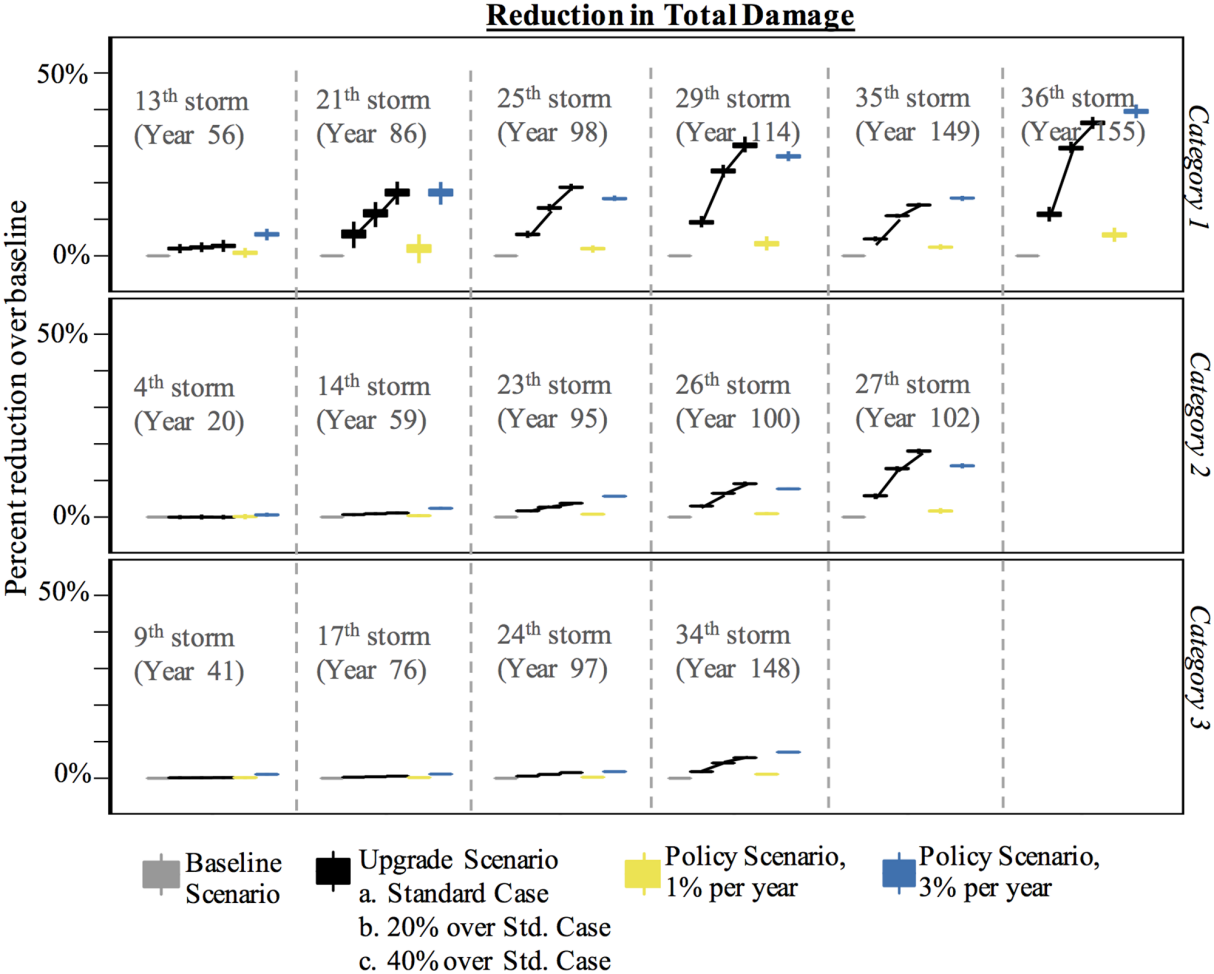
Percent reduction in total damage over baseline scenario for the upgrade, neighbor, and policy scenarios for Category 1, 2, and 3 storms.

**Fig 8:**
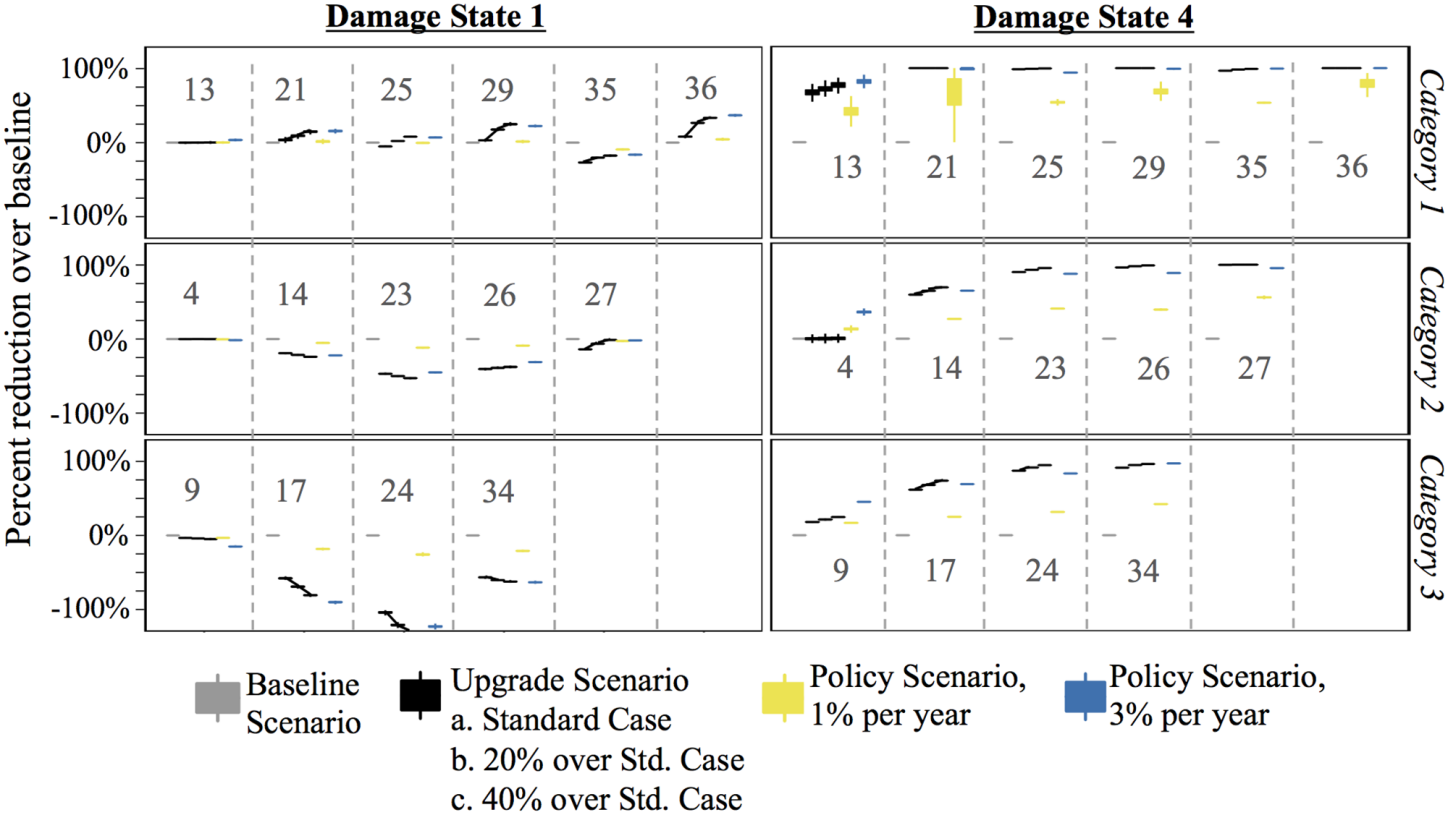
Percent reduction (or increase) in the number of houses in damage states 1 and 4 over baseline scenario for the upgrade, neighbor, and policy scenarios for Category 1, 2, and 3 storms. The dark gray numbers are the storm numbers. The year in which the storm occurred is found in Table 5.

Fig 7 shows the percent reduction in the total number of houses damaged over the baseline scenario for the upgrade, neighbor, and policy scenarios. The figure is divided into three parts: Category 1, Category 2, and Category 3 storms. Fig 8 accompanies Fig 7 and shows the percent reduction in damage states 1 and 4 for the same set of storms. By dividing the figures this way, we see how the expectation of mild and severe damage may change over time.

The fourth storm (in year 20) is the first Category 2 storm to impact the region. This storm damaged more than 80,000 houses, on average, in all scenarios except the 3% policy scenario. Of those houses to experience damage, approximately 30% experience severe damage (i.e., damage state 4). Fewer than 1,000 houses experience damage prior to this storm, so prior opportunities to mitigate are few in the upgrade and neighbor scenarios. In the 1% policy scenario, 82% of agents have yet to be selected to mitigate by year 20, so similarly few opportunities have arisen. Therefore, it is not surprising that these scenarios offer little reduction over baseline damage. The 3% policy scenario stands out. By year 20, 46% of agents have been selected to mitigate at least once and most of these improvements are to the roof (e.g., installing roof straps). While these improvements do little to protect the house from mild damage, they are effective at reducing the likelihood of severe damage from high winds—an important property in a wind environment like Miami. As such, the number of houses in damage state 4 decreases by almost 50% in the 3% policy scenario (Fig 8). Mostly, these are houses that otherwise would have been in damage state 4 but are now in damage states 2 or 3. This is significant because the structure of the house is mostly salvageable, hence reducing rebuilding costs. It is worth noting that the number of houses in damage state 3 *increases* 30% over baseline in the 3% policy scenario (not shown). This type of information—the distribution of the severity of damage—is helpful for city and regional planners. It informs them of the extent of regional damage after storms of varying intensity and how it could change over time.

By the first Category 1 storm—the 13^th^ storm to impact the region—at least 80% of houses have been damaged at least once (at any damage level) in the baseline and upgrade scenarios. Of those houses, 18% experience total loss. This offers agents opportunities to mitigate in the damage-driven mitigation scenarios and those that experience total devastation previously are more likely to make significant improvements. As a result of mitigation in earlier storms, the reduction in the number of houses in damage state 4 in the upgrade scenarios is noteworthy. The standard upgrade scenario reduces severe damage by nearly 70% and the more aggressive upgrade scenarios reduce severe damage by nearly 100% (Fig 8). In fact, by this storm the upgrade scenarios easily outpace the damage reduction offered by the 1% policy scenario.

**Fig 9:**
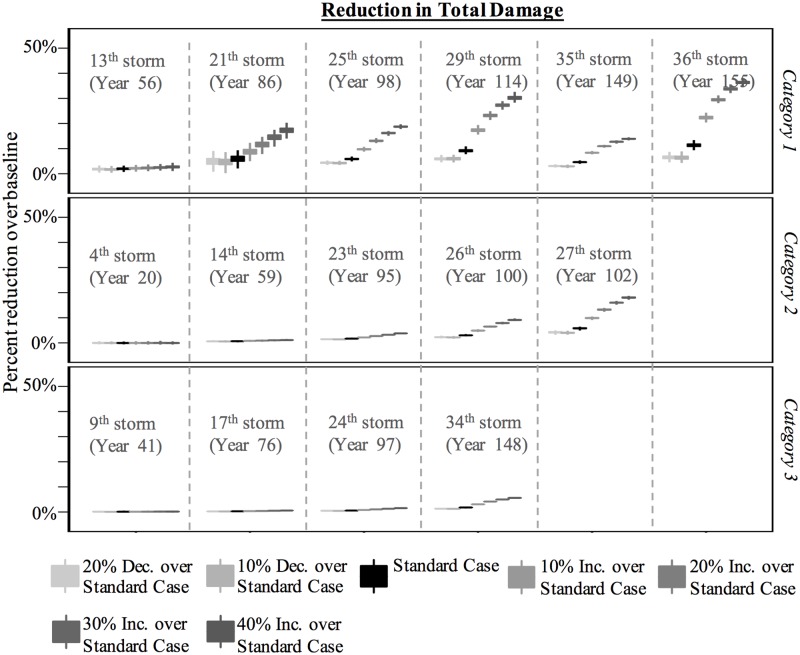
Sensitivity analysis for percent reduction in total damage (i.e., all damage states) over baseline scenario for the upgrade scenarios for Category 1, 2, and 3 storms.

The total reduction in damage continues to rise with each successive Category 1 storm (Fig 7). By the second Category 1 storm (the 21^st^ storm, which is the 6^th^ substantial storm), no house is expected to be severely damaged under the upgrade and policy scenarios. Further, the number of houses to experience mild damage is expected to decline (Fig 8). The level of damage reduction is heavily dependent on the likelihood of mitigation post-damage. Fig 9 shows a sensitivity analysis for the reduction in damage over baseline for different likelihoods of upgrading. The response is non-linear for Category 1 storms. For example, the largest marginal improvement is offered by the 10% increase over the standard case whereas the cases to offer the least marginal improvement are the 10% decrease and 40% increase over the standard case.

Interesting to note, the reduction in total damage in the 35^th^ storm is less than the reduction in total damage in the 29^th^ storm in the upgrade and policy scenarios. The 35^th^ storm was a large storm and affected more houses than most other Category 1 storms. Of the additional houses that were affected, many had not experienced damage in prior Category 1 storms—and only in Category 2 and 3 storms—so they had fewer opportunities to upgrade.

In contrast to Category 1 storms, the reduction in total damage in Category 2 and 3 storms is less. A couple of factors are at work here. First, many agents who experience damage in Category 2 and 3 storms do not experience damage in Category 1 and milder storms, in part because they live in less hazardous regions. Therefore, the number of opportunities they have to mitigate is fewer in damage-driven scenarios. The upgrades they do make, however, are more likely to reduce the risk of severe damage than mild damage. This is reflected the reduction of houses in damage state 4 and the increase of houses in damage state 1 in successive storms (Fig 8). However, the second and possibly more relevant reason for why Category 2 and 3 storms see minimal reduction in damage over the baseline scenario is because the winds are simply stronger, making any upgrades probabilistically less effective.

Another finding shown in Fig 7 is the relatively large variability in the reduction of damage over baseline for the 1% policy scenario in Category 1. In the upgrade scenario, the upgrades tend to occur in localized regions where storms are most likely to cause damage. In the policy scenarios, on the other hand, the upgrades are random, leading to greater variability in housing protection. This variability tends to decrease over time simply because there is a greater proportion of houses being upgraded. This variability is not apparent for Category 2 and 3 storms, because of the reduced effectiveness of upgrades, as noted above.

A neighbor scenario was also run for the Miami hurricane environment. The results, which are not shown, do not show an improvement over the upgrade scenarios. Similar to the Anne Arundel County hurricane environment, damage is often clustered and only a few houses without any damage exist within a cluster. Hence, if damage to neighboring houses spurs mitigation, it will likely only be relevant in areas where there is pronounced variability in damage.

#### Comparison of Anne Arundel County with Miami hurricane environments

The storms of the Miami hurricane environment are more frequent and intense than those of the Anne Arundel County hurricane environment. This allows us to compare how the environment can affect the outcomes over time under different scenarios and the effectiveness of different policy scenarios in particular. In the Anne Arundel County case, the 1% policy scenario is ineffective at reducing damage, in part because there is minimal damage to be had. In fact, the 1% policy scenario performs on par with even the most aggressive damage-driven upgrade scenarios, despite the fact that on average there is more than double the number of upgrades (Fig 6). This suggests that the expense of this degree of upgrades does not provide a comparable benefit. In contrast, the 1% policy in the Miami environment is ineffective at reducing damage compared to the other scenarios for a different reason; specifically because it provides too few opportunities to mitigate. Even in an upgrade scenario where the probability of upgrade given damage is relatively low (e.g., the 20% decrease over standard case), the number of upgrades that are made are more simply because there are more damage-causing storms.

The 3% policy scenario is effective at reducing damage in later storms in the Anne Arundel County case (Fig 4), but the type of damage that is reduced is mild. In this scenario, every house makes all possible upgrades, and the benefit for this massive improvement is the reduction of a few hundred broken windows. In contrast, the 3% policy scenario in the Miami hurricane environment is effective at reducing both total damage in Category I storms and severe damage in all storms. In fact, it outpaces the reduction in severe damage in storms 4 and 9 (Fig 8) compared to the upgrade scenarios, simply because there were fewer historical opportunities to mitigate in the damage driven scenarios. Hence, in regions with frequent and intense hurricanes, it could be effective to proactively encourage mitigation.

## Conclusions

In this work, we develop a simulation model to quantify the marginal effects of various behavioral scenarios and hazard environments on community vulnerability over time. This framework could allow regional planners and policy makers to identify potential policy solutions that are qualitatively robust against the deep uncertainty inherent with this problem. Through an illustrative example, we demonstrate how community vulnerability could evolve over time in Anne Arundel County given a range of decision rules. Using the behavioral rules that we test, we find that if the goal is to reduce the expected overall damage after a hurricane, blanket subsidies are usually more effective compared to damage-driven mitigation when the return period between hurricanes is long. We expect this, though, to come at great expense. However, when most agents upgrade by one level, they greatly reduce their potential for catastrophic losses in intense storms. Hence, there may be tremendous benefit for encouraging some minimal level of improvement early—because in reality, the year of arrival of the next severe storm is unknown. For environments that experience frequent and intense storms, we find that damage-driven mitigation can be as effective as blanket subsidies and that blanket subsidies exceed damage-driven mitigation only at fairly high rates of subsidy uptake. We also find a significant redistribution of damage states from state 4 towards state 1, indicating the effectiveness of the mitigation strategies. We remind the reader these results are derived from an illustrative example, and by no means do we consider the results to be robust enough to suggest changes to regional policy. A more comprehensive study that includes a wider range of behaviors would be required for that.

Many refinements could be added to this framework to that would enhance the insights from model runs. First, sophisticated learning and behavioral models for agents should be considered. How agents make decisions is complex and may depend on social norms, the perceived value and effectiveness of the upgrade, risk aversion, and cognitive biases. Improvement in behavioral modeling could help to answer questions like “how might information campaigns benefit a community?” or “in regions with tight social cohesion, how might social norms affect vulnerability?” Second, land use change should be considered [71]. This is especially important for quantifying how spatial-wealth disparities may compound over time due to repeated hazards. Third, hazards and large-scale community protection measures (e.g., levees, sea-walls) may be an important component to the framework. For example, levees may encourage people to remain in or move to more hazardous areas than they would otherwise, increasing the regional vulnerability in extreme events (e.g., [72]). Fourth, the role of insurance in our framework is not explicitly considered and ought to be. For more information on this class of work, we point the reader to Peng et al. [24], Carson et al. [73] and Kleindorfer and Kunreuther [74]. We acknowledge that insurance-homeowner interactions are important and the framework proposed in this paper could be expanded to consider them in future iterations.

## Appendix A. Mathematical description of illustrative example

We can describe the algorithm as a fully observable Markov decision processes. Following standard notation, we let

s_it_ = the state of house i after storm t

a_it_ = action by the homeowner of house i after storm t

π_k_(a|s) = probability of action a given state s under policy k

Pr(sʹ|s,a,tʹ) = transition probability of state sʹ after storm tʹ given the state s and action a immediately prior to this storm

For readability, we suppress subscripts in most of our description and use the standard prime superscript to denote the next time step, e.g., s denotes s_it_, sʹ denotes s_i,t+1_ and tʹ denotes t+1. In the main text, we consider only one major storm per year, but for the discussion of the mathematics below, it is not necessary to use this restriction.

Using this notation, we can now describe the mathematics of the illustrative example. We begin by describing the above constructs in more detail.

The state is given by a 5-tuple:

s = (r,d,x,y,h) in which

r = resistance level

d = damage level

x,y = location specified by longitude and latitude (fixed)

h = house type, e.g., two-story wood-framed house (fixed)

The fact that the location (x,y) is fixed implies that homeowners do not move, and the fact that h is fixed implies that houses are not entirely rebuilt, e.g., from a one-story to a two-story wood-framed house.

It is necessary to consider an intermediate state of the houses after homeowners repair and possibly upgrade their homes and prior to the next storm. We use the superscript 0 for this intermediate state. Hence, s is the state immediately after storm t, s^0^ is the state after repair/upgrade, and sʹ is the state after the next storm.

The action a produces two changes: the damage level d is reset to 0, meaning that the homeowner always repairs any damage, and the resistance level is set to a level greater or equal to its prior level:

a = (d^0^,r^0^) in which d^0^ = 0 and r^0^ > = r.

The action is stochastic, depending on the policy k. We describe the policies in more detail below, where we only show the probabilities for resistance level r^0^ since d^0^ = 0 with probability 1:

π_baseline_(a|s) corresponds to Pr(r^0^ = r) = 1

π_upgrade_(a|s) = Pr(r^0^|h,d) as specified in Table 3 in the main text and in the S2 Appendix.

π_neighbor_(a|s) depends on the damage state d:

π_neighbor_(a|s) = π_upgrade_(a|s) if d > 0, otherwise

π_neighbor_(a|s) corresponds to Pr(r^0^ = r+1) = p({d_jt_}, j ∈ neighborhood of house i) as specified in Table S2:24 and Pr(r^0^ = r) = 1 − Pr(r^0^ = r+1)

π_policy p_(a|s) also depends on damage state d:

π_policy p_(a|s) = π_upgrade_(a|s) if d > 0, otherwise

π_policy p_(a|s) corresponds to Pr(r^0^ = r+1) = p, Pr(r^0^ = r) = 1 − p

The storm t encompasses the entire region under study, and using the techniques describe in the methodology section of the main text, the properties of this storm are downscaled to the spatial scale of the parcels so that we obtain intensities:

I_it_ = intensity of the hazard of storm t on house i

We use these intensities to describe the stochastic effects of storm t on the intermediate state s^0^:

Pr(dʹ|I,h,r^0^) = fragility curve giving the probability of damage state dʹ for house h with resistance level r^0^ subjected to storm intensity I.

Given the above, the transition probability can be expressed as the following sum of the product of the fragility curve and the policy:

$$\text{Pr}\left(\text{s}\prime |\text{s,a,t}\prime \right)=\underset{{\text{r}}^{0}\geq \text{r}}{\sum }\text{Pr}\left(\text{d}\prime |{\text{I,h,r}}^{0}\right)\text{ Pr}\left({\text{r}}^{0}|\text{s}\right)=\underset{{\text{r}}^{0}\geq \text{r}}{\sum }\text{Pr}\left(\text{d}\prime |{\text{I,h,r}}^{0}\right)\;\pi_{\text{k}}\left({\text{d}}^{0}{\text{,r}}^{0}|\text{s}\right)$$

It is noted that the resistance r_i,t+1_ is assigned to be equal to r_it_^0^. This is necessary to maintain consistency in our Markov state description. It is not the true resistance of house i after storm t+1, but it is used as the reference resistance in the stochastic action a_i,t+1_ after storm t+1. For example, if house i is damaged by storm t+1, then under the upgrade policy,

$${\text{r}}_{\text{i},\text{t}+1}^{0}={\text{r}}_{\text{i},\text{t}+1}={\text{r}}_{\text{it}}^{0},$$

i.e., the resistance is set to be equal to the resistance after any repairs/upgrades performed after storm t. Also, as noted earlier in the definition of the policy, d^0^ = 1 with probability 1.

For all policies except for the neighbor policy, the transition probabilities above decoupled and can be evaluated for each house separately. For the neighbor policy, it is necessary to include the states of the neighboring houses. To make this last statement clearer, we use the subscript notation below:

$$\text{Pr}(s_{i,t+1}|s_{t},a_{it},\text{t}+1)=\sum_{r_{it}^{0}\geq r_{it}}\text{Pr}\left({\text{d}}_{i,t+1}|{\text{I}}_{it},{\text{h}}_{i},r_{it}^{0}\right)\pi_{\text{neighborhood}}\left(d_{it}^{0},r_{it}^{0}|s_{t}\right)$$

in which s_t_ is the state of all houses immediately after storm t.

If the storm intensity is also stochastic, then we would need one more summation over the range of possible intensities, multiplied by the probabilities of these intensities:

$$\text{Pr}\left(\text{s}\prime |\text{s,a,t}\prime \right)=\underset{I}{\sum }\underset{r^{0}\geq r}{\sum }\text{Pr}\left(\text{d}\prime |\text{I},\text{h},r^{0}\right)\pi_{k}\left(d^{0}\text{,}r^{0}|\text{s}\right)\text{ Pr}\left(\text{I}|\text{x},\text{y},\text{t}\prime \right)$$

## Supporting information

S1 Appendix Model details for illustrative example. An overview of the model, its structure, and implementation details.
(DOCX)

S2 Appendix Illustrative example inputs. A compilation of tables containing input data for the illustrative example. This includes building type characteristics, mitigation alternatives, and upgrade probabilities.
(DOCX)

S3 Appendix Code. The code used to run the model discussed in the illustrative example.
(DOCX)
