# Supplementary material for: Evolution of vulnerability of communities facing repeated hazards
Source: PLoS One. 2017 Sep 27;12(9):e0182719. doi: 10.1371/journal.pone.0182719 (PMC5617152; doi:10.1371/journal.pone.0182719)
Supplement: S1 Appendix — An overview of the model, its structure, and implementation details. (DOCX) [file pone.0182719.s001.docx]

**S1 Appendix. Model details for illustrative example**

The objective of this supporting appendix is to provide details about the model architecture and implementation. The model was developed in MATLAB, Version R2015b; no agent-based programming language was used. The reason for this is to allow for easier integration with hazard models that were already developed.

The architecture of the overall model is provided in Fig S1. The solid lines represent what is considered in the current work and the dotted lines represent aspects of what could feasibly be added. The basic flow of analysis steps is as follows:

1. Environment initialization
   1. A map of the region of interest is read into the simulation environment.
   2. Parcels are identified on the map.
   3. The houses, along with their structural properties, are placed on their corresponding parcels.
   4. Fragility curves are assigned to each house based on the parcel’s hazard, the building type, and current resistance level.
2. Run initialization
   1. A hazard mitigation scenario (baseline, upgrade, neighbor, policy 1% or policy 3%) is selected.
   2. A series of hazards are assigned over the time duration of interest.
   3. The initial resistance level is assigned to each house.
   4. Time is set to Year 1.
3. Hazard generation for each year
   1. A hazard is generated (e.g., a hurricane) at a spatial scale that encompasses the entire region under study.
   2. The properties of the hazard are downscaled to the spatial scale of the parcels (e.g., via [44]).
4. Damage generation for each house
   1. The downscaled hazard information is input into the fragility curve for the house to obtain the probabilities of various levels of damage
   2. A random uniform number generator is used to determine the simulated level of damage. This random value, along with the probabilities of various levels of damage from 4.a., report a house’s level of damage
5. Agent decision for each house
   1. The agent (homeowner) is given knowledge of the damage of his house and the damage of the houses of his neighbors.
   2. The agent makes a decision on mitigation depending on the scenario
      1. Baseline. If the house is damaged, the agent mitigates so that the house returns to its initial resistance level that was set in Year 1.
      2. Upgrade. The decision to mitigate is probabilistic and is a function of the damage the house sustains and its current resistance level. The probabilities of various upgrade in resistance is dependent on the damage state and are assigned using Table 3 in the main text (for one- and two-story wood-framed houses that are in resistance level 1 prior to the hazard) or the tables in the S2 Appendix (for all other houses and resistance levels).
      3. Neighbor.
         1. If the house is damaged, then the upgrade scenario is followed.
         2. Otherwise, the undamaged house is upgraded to the next highest resistance probability based on the probability that is dependent on the fraction of neighbors who experience damage.
      4. Policy 1%.
         1. If the house is damaged, then the baseline scenario is followed.
         2. Otherwise, there is a 1% probability that the undamaged house is selected for an upgrade to the next resistance level via a government subsidy.
      5. Policy 3%. This scenario is similar to the policy 1% scenario except that the 1% probability is replaced by 3%.
6. Steps 3-5 are repeated for the entire N-year hazard history (e.g., 162 years for the Anne Arundel illustrative example).
7. Steps 2-6 is repeated M times to obtain the averages and confidence intervals of damage states.

It is noted that the policy scenarios could be made more sophisticated by building scenarios that condition subsidies on expected future damage given the current building stock and hazard or total damage that was experienced after a hazard. Furthermore, it is feasible, in some select situations, that a large-scale government intervention could affect the hazard. For example, a government entity could build sea walls to reduce the likely height of surge. This, in return, could affect how agents perceive the risk.

**Fig A in S1 Appendix.** **Model Architecture.**

The dashed entities are not considered in the current work, but could be in the future.


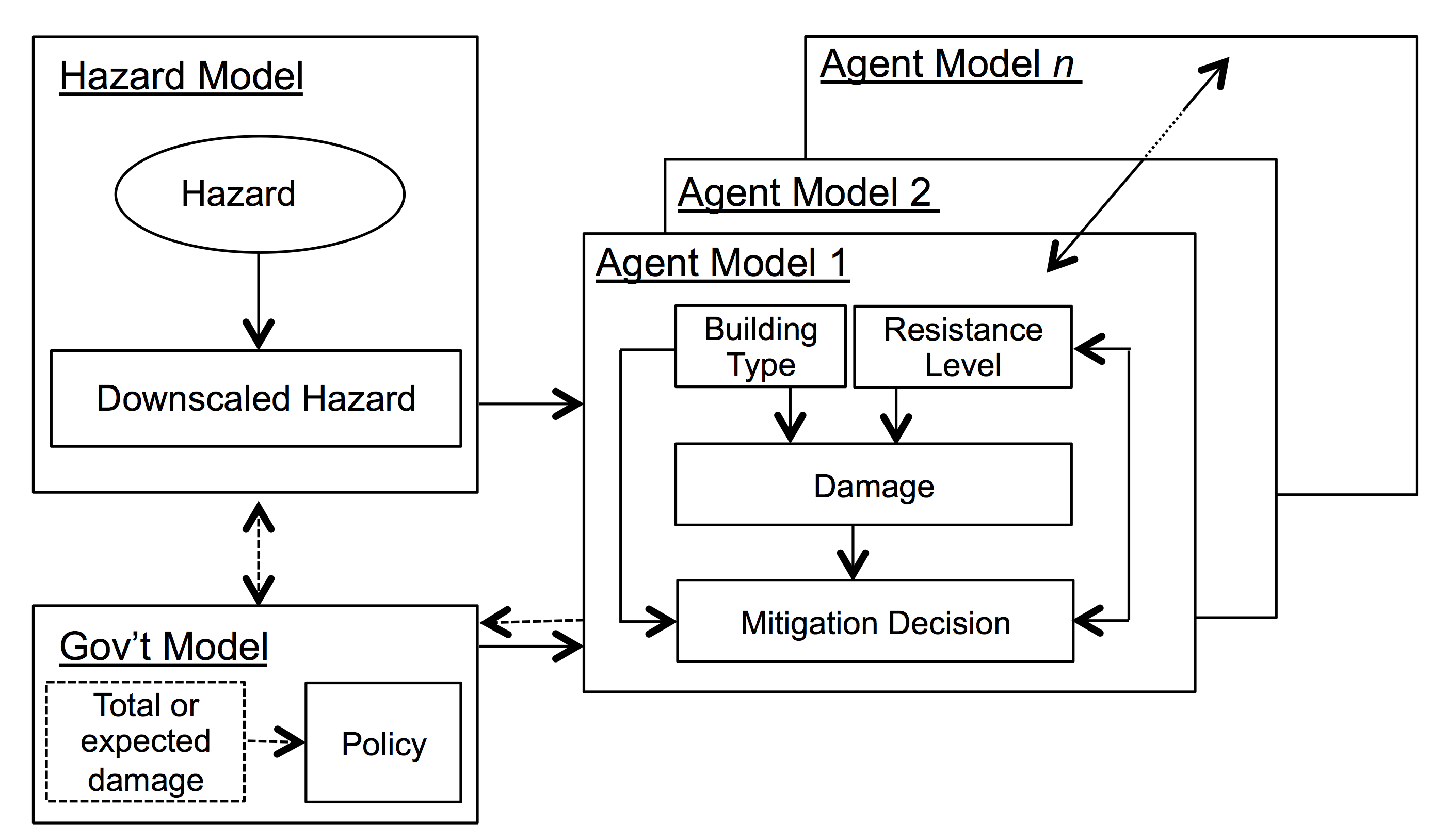


The model was run on a Xeon E5-2640 2.6GHz PC. Given the large number of agents, scenarios, sensitivity analyses, and replications, the model was massively parallelized [1]. Table S1 shows the approximate run times for each of the 4 mitigation scenarios. The units are CPU-hours and reflect the sum of the run times on each of the threads after 500 replications. The run times for a given scenario using different parameters are approximately equivalent. The neighbor scenario takes significantly longer to run due to neighbor-neighbor interactions.

**Table A in S1 Appendix.** **Run times (in hours) for each of the 4 scenarios.**

| **Baseline Scenario** | **Upgrade Scenario** | **Neighbor Scenario** | **Policy Scenario** |
| --- | --- | --- | --- |
| 25 | 42 | 412 | 38 |

**References**

1. Reilly AC, Staid A, Gao M, Guikema SD. Tutorial: Parallel Computing of Simulation Models for Risk Analysis. Risk Anal. 2016; 36(10):1844-1854. doi: 10.1111/risa.12565
