# Supplementary material for: Evolution of vulnerability of communities facing repeated hazards
Source: PLoS One. 2017 Sep 27;12(9):e0182719. doi: 10.1371/journal.pone.0182719 (PMC5617152; doi:10.1371/journal.pone.0182719)
Supplement: S2 Appendix — A compilation of tables containing input data for the illustrative example. This includes building type characteristics, mitigation alternatives, and upgrade probabilities. (DOCX) [file pone.0182719.s002.docx]

**S2 Appendix: Illustrative example inputs**

**Table A in S2 Appendix.** Building Type Characteristics

|  | Building Type | Building Characteristics | | |  | |  |
| --- | --- | --- | --- | --- | --- | --- | --- |
|  |  | Framing Material | Stand alone | No. Stories | | No. Buildings |  |
|  | **1** | Wood | Yes | 1 | | 53,810 |  |
|  | **2** | Wood | Yes | 2-3 | | 72,872 |  |
|  | **3** | Wood | No (townhouse) | 1 | | 2,147 |  |
|  | **4** | Wood | No (townhouse) | 2 | | 24,460 |  |
|  | **5** | Wood | No (townhouse) | 3 | | 6,292 |  |
|  | **6** | Unreinforced Masonry | Yes | 1 | | 636 |  |
|  | **7** | Unreinforced Masonry | Yes | 2 | | 215 |  |
|  | **8** | Unreinforced Masonry | No (townhouse) | 1 | | 945 |  |
|  | **9** | Unreinforced Masonry | No (townhouse) | 2 | | 17 |  |
|  | **10** | Concrete | Yes | 1-2 | | 650 |  |
|  | **11** | Mobile Homes | Yes | 1 | | 132 |  |
|  |  |  |  | **Total:** | | 162,176 |  |

**Table B in S2 Appendix.** Mitigation options for Building Types 1, 2, 3, 4, and 5 (one- and two-story, stand alone and townhouse, wood-framed houses)

|  | Resistance level | Building Characteristics | | | |  |
| --- | --- | --- | --- | --- | --- | --- |
|  |  | Roof Shape | Roof Deck Attachment | Roof-wall Connection | Shutters |  |
|  | **1** | Gable | 6d @ 6"/12" | Toe-nail | No |  |
|  | **2** | Gable | 6d @ 6"/12" | Strap | No |  |
|  | **3** | Gable | 6d @ 6"/12" | Strap | Yes |  |
|  | **4** | Gable | 8d @ 6"/12" | Strap | No |  |
|  | **5** | Gable | 8d @ 6"/12" | Strap | Yes |  |
|  | **6** | Hip | 8d @ 6"/12" | Strap | Yes |  |

**Table C in S2 Appendix.**Mitigation options for Building Types 6, 7, 8, and 9 (one- and two-story, stand alone and townhouse, unreinforced masonry houses)

|  | Resistance  level |  | | Building Characteristics | | | |  |
| --- | --- | --- | --- | --- | --- | --- | --- | --- |
|  |  | Roof Shape | Roof Deck Attachment | | Roof-wall Connection | Shutters | Reinforced Masonry |  |
|  | **1** | Gable | 6d @ 6"/12" | | Toe-nail | No | No |  |
|  | **2** | Gable | 6d @ 6"/12" | | Strap | No | No |  |
|  | **3** | Gable | 8d @ 6"/12" | | Strap | No | No |  |
|  | **4** | Gable | 6d @ 6"/12" | | Strap | No | Yes |  |
|  | **5** | Gable | 8d @ 6"/12" | | Strap | Yes | No |  |
|  | **6** | Gable | 8d @ 6"/12" | | Strap | No | Yes |  |
|  | **7** | Gable | 8d @ 6"/12" | | Strap | Yes | Yes |  |

**Table D in S2 Appendix.** Mitigation options for Building Type 10 (one- and two-story, stand alone and townhouse, concrete houses)

|  | Resistance level | Building Characteristics |  |
| --- | --- | --- | --- |
|  |  | Shutters |  |
|  | **1** | No |  |
|  | **2** | Yes |  |

**Table E in S2 Appendix.** Mitigation options for Building Types 11 (Mobile Homes)

|  | Resistance level | Building Characteristics | |  |
| --- | --- | --- | --- | --- |
|  |  | Shutters | Tie Downs |  |
|  | **1** | No | No |  |
|  | **2** | No | Yes |  |
|  | **3** | Yes | Yes |  |

**Table F in S2 Appendix.** Upgrade probabilities for Building Types 1-5 (one- and two-story, stand alone and townhouse, wood-framed houses) that are in resistance level 1 prior to the storm

|  | Resistance level after mitigation | Damage state after storm | | | |  |
| --- | --- | --- | --- | --- | --- | --- |
|  |  | 1 | 2 | 3 | 4 |  |
|  | **1** | 60% | 40% | 5% | 0 |  |
|  | **2** | 39% | 50% | 0 | 0 |  |
|  | **3** | 1% | 10% | 0 | 0 |  |
|  | **4** | 0 | 0 | 65% | 40% |  |
|  | **5** | 0 | 0 | 30% | 55% |  |
|  | **6** | 0 | 0 | 0 | 5% |  |

**Table G in S2 Appendix.**Upgrade probabilities for Building Types 1-5 (one- and two-story, stand alone and townhouse, wood-framed houses) that are in resistance level 2 prior to the storm

|  | Resistance level after mitigation | Damage state after storm | | | |  |
| --- | --- | --- | --- | --- | --- | --- |
|  |  | 1 | 2 | 3 | 4 |  |
|  | **1** | 0 | 0 | 0 | 0 |  |
|  | **2** | 70% | 45% | 10% | 0 |  |
|  | **3** | 10% | 15% | 10% | 0 |  |
|  | **4** | 20% | 40% | 55% | 30% |  |
|  | **5** | 0 | 0 | 24% | 65% |  |
|  | **6** | 0 | 0 | 1% | 5% |  |

**Table H in S2 Appendix.** Upgrade probabilities for Building Types 1-5 (one- and two-story, stand alone and townhouse, wood-framed houses) that are in resistance level 3 prior to the storm

|  | Resistance level after mitigation | Damage state after storm | | | |  |
| --- | --- | --- | --- | --- | --- | --- |
|  |  | 1 | 2 | 3 | 4 |  |
|  | **1** | 0 | 0 | 0 | 0 |  |
|  | **2** | 0 | 0 | 0 | 0 |  |
|  | **3** | 80% | 50% | 10% | 0 |  |
|  | **4** | 0 | 0 | 0 | 0 |  |
|  | **5** | 19% | 48% | 87% | 90% |  |
|  | **6** | 1% | 2% | 3% | 10% |  |

**Table I in S2 Appendix.** Upgrade probabilities for Building Types 1-5 (one- and two-story, stand alone and townhouse, wood-framed houses) that are in resistance level 4 prior to the storm

|  | Resistance level after mitigation | Damage state after storm | | | |  |
| --- | --- | --- | --- | --- | --- | --- |
|  |  | 1 | 2 | 3 | 4 |  |
|  | **1** | 0 | 0 | 0 | 0 |  |
|  | **2** | 0 | 0 | 0 | 0 |  |
|  | **3** | 0 | 0 | 0 | 0 |  |
|  | **4** | 75% | 60% | 35% | 20% |  |
|  | **5** | 24% | 38% | 60% | 70% |  |
|  | **6** | 1% | 2% | 5% | 10% |  |

**Table J in S2 Appendix.** Upgrade probabilities for Building Types 1-5 (one- and two-story, stand alone and townhouse, wood-framed houses) that are in resistance level 5 prior to the storm

|  | Resistance level after mitigation | Damage state after storm | | | |  |
| --- | --- | --- | --- | --- | --- | --- |
|  |  | 1 | 2 | 3 | 4 |  |
|  | **1** | 0 | 0 | 0 | 0 |  |
|  | **2** | 0 | 0 | 0 | 0 |  |
|  | **3** | 0 | 0 | 0 | 0 |  |
|  | **4** | 0 | 0 | 0 | 0 |  |
|  | **5** | 99% | 95% | 93% | 80% |  |
|  | **6** | 1% | 5% | 7% | 20% |  |

**Table K in S2 Appendix.** Upgrade probabilities for Building Types 1-5 (one- and two-story, stand alone and townhouse, wood-framed houses) that are in resistance level 5 prior to the storm

|  | Resistance level after mitigation | Damage state after storm | | | |  |
| --- | --- | --- | --- | --- | --- | --- |
|  |  | 1 | 2 | 3 | 4 |  |
|  | **1** | 0 | 0 | 0 | 0 |  |
|  | **2** | 0 | 0 | 0 | 0 |  |
|  | **3** | 0 | 0 | 0 | 0 |  |
|  | **4** | 0 | 0 | 0 | 0 |  |
|  | **5** | 0 | 0 | 0 | 0 |  |
|  | **6** | 100% | 100% | 100% | 100% |  |

**Table L in S2 Appendix.** Upgrade probabilities for Building Types 6-9 (one- and two-story, stand alone and townhouse, unreinforced masonry houses) that are in resistance level 1 prior to the storm

|  | Resistance level after mitigation | Damage state after storm | | | |  |
| --- | --- | --- | --- | --- | --- | --- |
|  |  | 1 | 2 | 3 | 4 |  |
|  | **1** | 60% | 40% | 30% | 0 |  |
|  | **2** | 38% | 53% | 0 | 0 |  |
|  | **3** | 1% | 5% | 0 | 0 |  |
|  | **4** | 1% | 2% | 35% | 0 |  |
|  | **5** | 0 | 0 | 0 | 0 |  |
|  | **6** | 0 | 0 | 35% | 70% |  |
|  | **7** | 0 | 0 | 0 | 30% |  |

**Table M in S2 Appendix.** Upgrade probabilities for Building Types 6-9 (one- and two-story, stand alone and townhouse, unreinforced masonry houses) that are in resistance level 2 prior to the storm

|  | Resistance level after mitigation | Damage state after storm | | | |  |
| --- | --- | --- | --- | --- | --- | --- |
|  |  | 1 | 2 | 3 | 4 |  |
|  | **1** | 0 | 0 | 0 | 0 |  |
|  | **2** | 75% | 70% | 25% | 0 |  |
|  | **3** | 20% | 20% | 20% | 0 |  |
|  | **4** | 5% | 10% | 45% | 0 |  |
|  | **5** | 0 | 0 | 10% | 0 |  |
|  | **6** | 0 | 0 | 0 | 70% |  |
|  | **7** | 0 | 0 | 0 | 30% |  |

**Table N in S2 Appendix.** Upgrade probabilities for Building Types 6-9 (one- and two-story, stand alone and townhouse, unreinforced masonry houses) that are in resistance level 3 prior to the storm

|  | Resistance level after mitigation | Damage state after storm | | | |  |
| --- | --- | --- | --- | --- | --- | --- |
|  |  | 1 | 2 | 3 | 4 |  |
|  | **1** | 0 | 0 | 0 | 0 |  |
|  | **2** | 0 | 0 | 0 | 0 |  |
|  | **3** | 60% | 55% | 45% | 0 |  |
|  | **4** | 0 | 0 | 0 | 0 |  |
|  | **5** | 35% | 40% | 45% | 0 |  |
|  | **6** | 0 | 0 | 0 | 70% |  |
|  | **7** | 5% | 5% | 10% | 30% |  |

**Table O in S2 Appendix.** Upgrade probabilities for Building Types 6-9 (one- and two-story, stand alone and townhouse, unreinforced masonry houses) that are in resistance level 4 prior to the storm

|  | Resistance level after mitigation | Damage state after storm | | | |  |
| --- | --- | --- | --- | --- | --- | --- |
|  |  | 1 | 2 | 3 | 4 |  |
|  | **1** | 0 | 0 | 0 | 0 |  |
|  | **2** | 0 | 0 | 0 | 0 |  |
|  | **3** | 0 | 0 | 0 | 0 |  |
|  | **4** | 75% | 55% | 10% | 0 |  |
|  | **5** | 0 | 0 | 0 | 0 |  |
|  | **6** | 20% | 40% | 50% | 60% |  |
|  | **7** | 5% | 5% | 40% | 40% |  |

**Table P in S2 Appendix.** Upgrade probabilities for Building Types 6-9 (one- and two-story, stand alone and townhouse, unreinforced masonry houses) that are in resistance level 5 prior to the storm

|  | Resistance level after mitigation | Damage state after storm | | | |  |
| --- | --- | --- | --- | --- | --- | --- |
|  |  | 1 | 2 | 3 | 4 |  |
|  | **1** | 0 | 0 | 0 | 0 |  |
|  | **2** | 0 | 0 | 0 | 0 |  |
|  | **3** | 0 | 0 | 0 | 0 |  |
|  | **4** | 0 | 0 | 0 | 0 |  |
|  | **5** | 90% | 80% | 60% | 0 |  |
|  | **6** | 0 | 0 | 0 | 20% |  |
|  | **7** | 10% | 20% | 40% | 80% |  |

**Table Q in S2 Appendix.** Upgrade probabilities for Building Types 6-9 (one- and two-story, stand alone and townhouse, unreinforced masonry houses) that are in resistance level 6 prior to the storm

|  | Resistance level after mitigation | Damage state after storm | | | |  |
| --- | --- | --- | --- | --- | --- | --- |
|  |  | 1 | 2 | 3 | 4 |  |
|  | **1** | 0 | 0 | 0 | 0 |  |
|  | **2** | 0 | 0 | 0 | 0 |  |
|  | **3** | 0 | 0 | 0 | 0 |  |
|  | **4** | 0 | 0 | 0 | 0 |  |
|  | **5** | 0 | 0 | 0 | 0 |  |
|  | **6** | 75% | 50% | 25% | 30% |  |
|  | **7** | 25% | 50% | 75% | 70% |  |

**Table R in S2 Appendix.** Upgrade probabilities for Building Types 6-9 (one- and two-story, stand alone and townhouse, unreinforced masonry houses) that are in resistance level 7 prior to the storm

|  | Resistance level after mitigation | Damage state after storm | | | |  |
| --- | --- | --- | --- | --- | --- | --- |
|  |  | 1 | 2 | 3 | 4 |  |
|  | **1** | 0 | 0 | 0 | 0 |  |
|  | **2** | 0 | 0 | 0 | 0 |  |
|  | **3** | 0 | 0 | 0 | 0 |  |
|  | **4** | 0 | 0 | 0 | 0 |  |
|  | **5** | 0 | 0 | 0 | 0 |  |
|  | **6** | 0 | 0 | 0 | 0 |  |
|  | **7** | 100% | 100% | 100% | 100% |  |

**Table S in S2 Appendix.** Upgrade probabilities for Building Type 10 (one- and two-story, stand alone and townhouse, concrete houses) that are in resistance level 1 prior to the storm

|  | Resistance level after mitigation | Damage state after storm | | | |  |
| --- | --- | --- | --- | --- | --- | --- |
|  |  | 1 | 2 | 3 | 4 |  |
|  | **1** | 95% | 90% | 75% | 40% |  |
|  | **2** | 5% | 10% | 25% | 60% |  |

**Table T in S2 Appendix.** Upgrade probabilities for Building Type 10 (one- and two-story, stand alone and townhouse, concrete houses) that are in resistance level 2 prior to the storm

|  | Resistance level after mitigation | Damage state after storm | | | |  |
| --- | --- | --- | --- | --- | --- | --- |
|  |  | 1 | 2 | 3 | 4 |  |
|  | **1** | 0 | 0 | 0 | 0 |  |
|  | **2** | 100% | 100% | 100% | 100% |  |

**Table U in S2 Appendix.** Upgrade probabilities for Building Type 11 (mobile homes) that are in resistance level 1 prior to the storm

|  | Resistance level after mitigation | Damage state after storm | | | |  |
| --- | --- | --- | --- | --- | --- | --- |
|  |  | 1 | 2 | 3 | 4 |  |
|  | **1** | 70% | 60% | 50% | 10% |  |
|  | **2** | 20% | 30% | 25% | 60% |  |
|  | **3** | 10% | 10% | 25% | 30% |  |

**Table V in S2 Appendix.** Upgrade probabilities for Building Type 11 (mobile homes) that are in resistance level 2 prior to the storm

|  | Resistance level after mitigation | Damage state after storm | | | |  |
| --- | --- | --- | --- | --- | --- | --- |
|  |  | 1 | 2 | 3 | 4 |  |
|  | **1** | 0 | 0 | 0 | 0 |  |
|  | **2** | 80% | 60% | 50% | 30% |  |
|  | **3** | 20% | 40% | 50% | 70% |  |

**Table W in S2 Appendix.** Upgrade probabilities for Building Type 11 (mobile homes) that are in resistance level 3 prior to the storm

|  | Resistance level after mitigation | Damage state after storm | | | |  |
| --- | --- | --- | --- | --- | --- | --- |
|  |  | 1 | 2 | 3 | 4 |  |
|  | **1** | 0 | 0 | 0 | 0 |  |
|  | **2** | 0 | 0 | 0 | 0 |  |
|  | **3** | 100% | 100% | 100% | 100% |  |

**Table X in S2 Appendix.** Upgrade probabilities for homeowners in neighbor case

|  | Upgrade 1 Resistance Level if: | Fraction of neighbors within 75m to experience damage is greater than | | | | | |
| --- | --- | --- | --- | --- | --- | --- | --- |
|  |  | 10% | 20% | 30% | 40% | 50% |  |
