# Supplementary material for: Evolution of vulnerability of communities facing repeated hazards
Source: PLoS One. 2017 Sep 27;12(9):e0182719. doi: 10.1371/journal.pone.0182719 (PMC5617152; doi:10.1371/journal.pone.0182719)
Supplement: S3 Appendix — The code used to run the model discussed in the illustrative example. (DOCX) [file pone.0182719.s003.docx]

**S3 Appendix: Code**

**List of .m files needed to run ABM**

(1) Hazard_SEES_ABM.m

(2) Return_Damage_Initial.m

(3) findDamageGivenWS.m

(4) Mitigation_Scenario2_Upgrade.m

(5) Mitigation_Scenario3_Neighbor_Mod.m

(6) Mitigation_Scenario4_Policy.m

(7) Returns_NewResistanceLevel.m

(8) CountNumUpgrades_inReplication.m

(9) save_data.m

***************************

**(1) Hazard_SEES_ABM.m**

function Hazard_SEES_ABM(caseNo, city, StormWindSpeed, percentHouseGivenSubsidy, ...

Upgrade_Sensitivity_factor, threshold_Neigh, numReplications, ProbMit, FragilityCurve,... Neigh_List, StormsPerYear, numHurr, Parcel_Input, numYear, ThresholdElevateMech, ... ThresholdElevateHouse, MinMechProb, MinRaiseProb, parallel, prior, Cost_route, ...

damage_prob_new, Upgrade_route)

%****Program Description*******

%this is the master file that, for each replication and year, assesses

%damage (wind) for each parcel, determines which mitigation will

%happen, and then saves the results

%********Inputs*****************

%numYear = 162 %size: 1 x 1; may be unnecessary if using complete historical record

%numReplications = the number of replications.

%Parcel_Input: Information on the parcel characteristics. Col1: Parcel ID; Col2:

%    Superseeded? NaN or No which points to new Parcel ID(s);

% Col3: Building Type (1-17)

% Col4: Resitance Level (1 - lowest through 7 - highest);

%    Col5: List of Years Damaged

%StormWindSpeed peak 3s windgust of storm j for household i,

%     size: num households + 1 x num storms. Row 1 represents parcel ID.  Load this

%     if using historical record.

%StormsPerYear    % col1: year;  col2: # storms; col3: 0 if no storms caused damage, ID #

%     of first storm in year i otherwise; col4: 0 if no storms caused damage,

%     ID# of last storm in year i otherwise is now year_final

%ProbMit: A probability matrix of upgrade likelihood to a higher

%     resistance level after a strom and in DS #: size: no resistance level

%     (to upgrade to) x no damage states x no resistance levels (before

%     storm) USE 'Mit_Prob' (4 x 1 cell)

%FragilityCurve: the fragility curves, size: 41 x 17 x 7 x 4 (check), 'WFC'

%     in Laiyin's code

%CaseNo: 1, 2, 3, or 4. Size = 1 x 1. if =2, then upgrade case, if =3 then

%   upgrade + neighbor case, if =4 then tax subsidy case

%City: 'AA','Miami'

%Upgrade_Sensitivity_factor: the amount to adjust upgrade probabilities by

%   for sensitivity analysis. e.g., 0.1, -0.25 --> 10% inc, 25% dec

%threshold_Neigh: The threshold of the fraction of neighbors (houses within 75m)

%    who are damaged (at Damage State 2 or above) that above which a parcel will

%    upgrade one resistance level

%Neigh_List: The list of neighbors (within 75m) for each parcel.

%   Size: num parcels x max(number of neighbors)

%%%%%%%%%%%%%%%%%%%%%%%%%%%%%%%%%%%%%%%%%%%%%%%%%%%%%%%%

%     Parallelization

%%%%%%%%%%%%%%%%%%%%%%%%%%%%%%%%%%%%%%%%%%%%%%%%%%%%%%%%

if parallel

    %If not inside a PBS job, use 2 processors

    if isempty(getenv('PBS_NP'))

        NP = 2;

    else

        NP = str2double(getenv('PBS_NP'));

    end

    myPool = parpool('current',NP);

end

%total number of years with at least one storm

numYearWStorm = sum(StormsPerYear(:,3)>0); %36 initially for Miami case

numStorm = max(StormsPerYear(:,4)); %47 initially for Miami case

numParcelsInitial = size(Parcel_Input,1);

hurricane_count = zeros(numParcelsInitial,7);

parfor replication = 1:numReplications  %No. replications

    StormsPerYear_Loop=StormsPerYear;

    StormWindSpeed_Loop = StormWindSpeed;

    fprintf(1,'Replication: %d \n',replication);

    Parcel = Parcel_Input; %Initialize Parcel data

    %initialize building stock for replication

    Parcel = Initialize_RLofBuildingStock(Parcel);

    Parcel_forUpgadeCount = Parcel(:,[1 2 3 4]);

    Damage_storm = zeros(numParcelsInitial,12,numStorm); %records parcel damage in

        %storm x in year 'year.' There could be multiple storms in year 'year'

    Damage_year = zeros(numParcelsInitial,12,numYearWStorm); %records parcel damage in

        %year 'year.' If there are multiple storms in year 'year' that damage the parcel, the worst

        %damage state is used.

    Upgrade_year_tracker = zeros(numParcelsInitial,11);

    Upgrade_year_tracker(:,1) = Parcel(:,1);

    Upgrade_year_tracker(:,2) = 1;

    Upgrade_year_tracker(:,3) = Parcel(:,4);

    yearNext = 0;

    for year = 1:numYear   %No. years of historical hurricane history

        %%%%%%%%%%%%%%%%%%%%%%%%%%%%%%%%%%%%%%%%%%%%%%%%%%%%%%%%

        %

        %           Damage

        %

        %%%%%%%%%%%%%%%%%%%%%%%%%%%%%%%%%%%%%%%%%%%%%%%%%%%%%%%%

        %Determine how many storms in year j cause damage

        %Use lookup table for no. storms, windspeeds

        %Make a list the storms in 'year' that cause damage

        if StormsPerYear_Loop(year,3) > 0

            yearNext = yearNext+1; %records num years with storm; max  should be 36

            StormIDsInYear = StormsPerYear_Loop(year,3):1:StormsPerYear_Loop(year,4); %List of stormIDs

            %that occur in 'year'

        else

            %no storm occured in 'year' that brought damage

            StormIDsInYear=NaN;

        end

        if ~isnan(StormIDsInYear) %if there is at least one storm

            %Loop determines damage to each house from storm k

            for storm = 1:length(StormIDsInYear)  %for each storm that produces damage in year j

                Damage_storm(:,:,StormIDsInYear(storm)) = Return_Damage_Initial(Parcel,year);

                %WIND DAMAGE

                Damage_Internal = findDamageGivenWS(Parcel, FragilityCurve, ...

                    StormWindSpeed_Loop([1;StormIDsInYear(storm)+1],:), year);

                Damage_storm(:,7:8,StormIDsInYear(storm)) = Damage_Internal;

                %%%%%%%%%%%%%%%%%%%%%%%%%%%%

            end

        %%%%%%%%%%%%%%%%%%%%%%%%%%%%%%%%%%%%%%%%%%%%%%%%%%%%%%%%

        %           2+ Storms in a year

        %%%%%%%%%%%%%%%%%%%%%%%%%%%%%%%%%%%%%%%%%%%%%%%%%%%%%%%%

            %POPULATE Damage_year: If House is damaged 2+ times in

            %   'year', record the worse of the damage

            if length(StormIDsInYear) > 1 % if more than 1 storm

                %for each parcel in Damage_storm in indices 'StormIDsInYear', find the

                %     max damage state (col 7) and the corresponding WS (col 8 and 9)

Damage_temp = [Damage_storm(:,1:6,StormIDsInYear(1)) ... zeros(size(Damage_storm(:,1),1),6)];

                for index = 1:size(Damage_storm,1) %for each parcel

                    [maxDS, indexMaxDS] = max(Damage_storm(index,7,StormIDsInYear)); %find max damage

                    %and from which storm

Damage_temp(index,7:8) = [maxDS ... Damage_storm(index,8,StormIDsInYear(indexMaxDS(1)))];

                end

                Damage_year(:,:,yearNext)=Damage_temp;

            else %There is only one storm in year 'year'; the list of building that are damaged

                Damage_year(:,:,yearNext)=Damage_storm(:,:,StormIDsInYear);

            end

        end

        %%%%%%%%%%%%%%%%%%%%%%%%%%%%%%%%%%%%%%%%%%%%%%%%%%%%%%%%

        %

        %           Mitigation

        %

        %%%%%%%%%%%%%%%%%%%%%%%%%%%%%%%%%%%%%%%%%%%%%%%%%%%%%%%%

        %Case 1 (return to original resistance level)

        %Damage is recorded above; no need to change resistance level in Parcel

        %Case 2 (probability of some upgrade)

        %If parcel experienced damage at some point during 'year', they will upgrade

        %     with some probability

        if caseNo==2

            if ~isnan(StormIDsInYear)

                [Parcel, damageTemp,Upgrade_year_tracker] = ...

Mitigation_Scenario2_Upgrade(Damage_year(:,:,yearNext), Parcel, ...

ProbMit, Upgrade_year_tracker, year);

                Damage_year(:,:,yearNext) = damageTemp;

            end

        end

        %Case 3 (neighborhood effect)

        if caseNo==3

            if ~isnan(StormIDsInYear)

                [Parcel, damageTemp,Upgrade_year_tracker] = ...

                Mitigation_Scenario2_Upgrade(Damage_year(:,:,yearNext), Parcel, ProbMit, ... Upgrade_year_tracker,year);

Damage_year(:,:,yearNext) = damageTemp;

[Parcel,damageNeighTemp, Upgrade_year_tracker] = ...

Mitigation_Scenario3_Neighbor_Mod(Parcel, Damage_year(:,:,yearNext), ...

threshold_Neigh, year, Upgrade_year_tracker, Neigh_List);

Damage_year(:,:,yearNext) = damageNeighTemp;

            end

        end

        %Case 4 (tax incentive - x% of home upgrade to next resistance level)

        if caseNo==4

            [Parcel, Upgrade_year_tracker] = Mitigation_Scenario4_Policy(Parcel, ... percentHouseGivenSubsidy, year,Upgrade_year_tracker);

    end

    %for all cases, count number of upgrades for each parcel

    NoUpgradesMaster = CountNumUpgrades_inReplication(Parcel, Parcel_Input, Parcel_forUpgadeCount);

    %add Parcel and Damage for replication i to master Parcel and Damage

    Damage_Master_Year= Damage_year;

    Upgrade_year_tracker_Rep = Upgrade_year_tracker;

    %Save data

    save_data(replication, city, caseNo, Damage_Master_Year, Upgrade_year_tracker_Rep, ...

        NoUpgradesMaster, Upgrade_Sensitivity_factor, threshold_Neigh, percentHouseGivenSubsidy)

    %organize damage data and save

    Extract_Output_Data(city, numHurr, Damage_Master_Year, replication,...

        caseNo, Upgrade_Sensitivity_factor, percentHouseGivenSubsidy,...

        threshold_Neigh);

end

if parallel

    delete(myPool);

end

consolidateOutputData_fromBatches(city, numHurr, numReplications, caseNo, ...

Upgrade_Sensitivity_factor, percentHouseGivenSubsidy, threshold_Neigh)

end

***************************

**(2) Return_Damage_Initial.m**

function Damage_Internal = Return_Damage_Initial(Parcel,Year)

Damage_Internal = zeros(size(Parcel,1),12);

Damage_Internal(:,1) = Year;

Damage_Internal(:,2:5) = Parcel(:,1:4);

Damage_Internal(:,6) = Parcel(:,4);   %Resistance level new

end

***************************

**(3) findDamageGivenWS.m**

function [Damage_Internal] = findDamageGivenWS(Parcel, Fragility_Curves, Windspeed, Year)

% This function probabilistically assigns damage states (0, 1, 2, 3, or 4) given wind speed for all parcels

%Called by: Hazard_SEES_ABM.m

%%%%%%%OUTPUT%%%%%%%%%%%%%

%Damage_Internal: a matrix (double) that lists all parcels and their damage

% if any in 'Year'; size: noParcel(active+old) x 9 matrix (double)

%     Col1: year

%     Col2: Parcel ID

%     Col3: Parcel active? NaN if yes or ## if superceeded

% Col4: Building Type (1-11),

%     Col5: Resistance Level at START of year

%     Col6: Resistance Level at END of year

%     Col7: Max Damage State (1-4) in year

%     Col8: Windspeed that produced damage in year

%Define the windspeed for fragility curves

AllWindspeed= linspace(50,250,41);

%This is a unit converter; 1.151 is knots --> mph

WS_converter = 1.151;

%inialize output 'Damage_Interal'; is the number of Parcels (active + old)

Damage_Internal = zeros(size(Parcel,1),8);

Damage_Internal(:,1) = Year;

Damage_Internal(:,2:5) = Parcel(:,1:4);

Damage_Internal(:,6) = Parcel(:,4);   %Resistance level new (same as old until updated)

%for each parcel (given its building type and resistance level,

%    determine if there is damage and the severity of damage

for BuildingType = 1:11  %11 building types

    for ResistanceLevel = 1:size(Fragility_Curves{BuildingType,1},1)

        %list of active parcels with building type i and resistance level j

        Parcel_BuildingResistance_IDs =  Parcel(Parcel(:,3)==...

            BuildingType & Parcel(:,4)==ResistanceLevel & isnan(Parcel(:,2)),1);

        %What are the rows in Parcel that correspond to the Parcel_BuildingResistance_IDs list?

        Parcel_BuildingResistance_IDs_index = find(Parcel(:,3)==...

            BuildingType&Parcel(:,4)==ResistanceLevel&isnan(Parcel(:,2))); %the indices in

        %Parcel that contain parcels with BT, RL, and that are active

        %setOfColumns: find columns in Windspeed that refer to Parcel_BuildingResistance_IDs

        setOfColumns = ismember(Windspeed(1,:),Parcel_BuildingResistance_IDs);

%ParcelWS: Array of Parcel windspeed, in mph (for 'BuildingType' and 'ResistanceLevel')

        ParcelWS = Windspeed(2, setOfColumns)*WS_converter;

        %Prob of DS_X given windspeed, building type, and building resistance level

        ProbDamageState_1= interp1(AllWindspeed, ...

Fragility_Curves{BuildingType,1}(ResistanceLevel,:,1), ParcelWS);

ProbDamageState_2= interp1(AllWindspeed, ... Fragility_Curves{BuildingType,1}(ResistanceLevel,:,2), ParcelWS);

ProbDamageState_3= interp1(AllWindspeed, ... Fragility_Curves{BuildingType,1}(ResistanceLevel,:,3), ParcelWS);

ProbDamageState_4= interp1(AllWindspeed, ...

Fragility_Curves{BuildingType,1}(ResistanceLevel,:,4), ParcelWS);

        %if isnan, convert Probability to 0

        ProbDamageState_1(1,isnan(ProbDamageState_1(1,:)))=0;

        ProbDamageState_2(1,isnan(ProbDamageState_2(1,:)))=0;

        ProbDamageState_3(1,isnan(ProbDamageState_3(1,:)))=0;

        ProbDamageState_4(1,isnan(ProbDamageState_4(1,:)))=0;

        %order ProbDamageState 1 through 4; This makes sure that the Prob of

        %being in each of the damage states goes in descending order

        temp = [ones(1,size(ProbDamageState_1,2)); ProbDamageState_1;ProbDamageState_2;...

            ProbDamageState_3;ProbDamageState_4;zeros(1,size(ProbDamageState_1,2))];

[~, DamageIndices] = sort(temp,'descend');

        ProbDamageState_4(1,DamageIndices(5,:) ~= 5)=0;

        temp = [ones(1,size(ProbDamageState_1,2)); ProbDamageState_1;ProbDamageState_2;...

            ProbDamageState_3;ProbDamageState_4;zeros(1,size(ProbDamageState_1,2))];

        ProbDamageState = sort(temp,'descend');

        RandNo = rand(1,size(ProbDamageState_1,2));

        DS = zeros(1,size(ProbDamageState_1,2)); %No buildings of BuildingType_i and ResLevel_j,

%for each building with ResLevelj and BuildTypei and active

        for k= 1:size(ProbDamageState_1,2)

            DS(1,k) = find(ProbDamageState(:,k)>RandNo(1,k),1,'last')-1;

            Parcel_Index = Parcel_BuildingResistance_IDs_index(k,1);

            %update Damage List, Parcel List if there is damage

            Damage_Internal(Parcel_Index,7:8) = [DS(1,k) ParcelWS(1,k)];

        end

    end

end

Damage_Internal = Damage_Internal(:,7:8);

end

***************************

**(4) Mitigation_Scenario2_Upgrade.m**

function [Parcel, Damage_year,Upgrade_year_tracker] = Mitigation_Scenario2_Upgrade(Damage_year, Parcel, ProbMit,Upgrade_year_tracker,year)

% This function is for mitigation Option 2 - when a house is damaged after a

% storm, there is some likelihood that it will increase its resistance level

% given its damage state and the resistance level in which it began

%

%Called by: Hazard_SEES_ABM.m

for i = 1:size(Damage_year,1) %number of Parcels

      if isnan(Damage_year(i,3))

            DamageLevel = Damage_year(i,7);  %House Damage Level

            if DamageLevel>=1

                  %House Resistance Level before storm

                  ResistanceLevelCurrent  = Damage_year(i,5);

                  BuildingType = Damage_year(i,4);

                  if BuildingType<6

                      Type = 1;

                  elseif BuildingType<10

                      Type = 2;

                  elseif BuildingType==10

                      Type = 3;

                  else

                      Type = 4;

                  end

%Probability of upgradeS given resistance

                  Prob = ProbMit{Type,1}(:,DamageLevel, ResistanceLevelCurrent);

                  Prob = cumsum(Prob);

                  RandNo = rand();

                  %find index in Prob where RandNo lies <- this the is new

                  %resistance level

                  temp= RandNo-Prob;

                  NewResistanceLevel = find(temp<=0,1);

                  if BuildingType==10 && NewResistanceLevel>2

                      NewResistanceLevel=2;

                  end

                  %Update Parcel information

                  Damage_year(i,6) = NewResistanceLevel; %col 6: new resistance level

                  Parcel(i,4) = NewResistanceLevel;

                  temp2 = find(Upgrade_year_tracker(Parcel(i,1),:)>0);

                  if temp2(end) < 10 && NewResistanceLevel > ResistanceLevelCurrent

                      Upgrade_year_tracker(Parcel(i,1),temp2(end)+1) = year; %year

                      Upgrade_year_tracker(Parcel(i,1),temp2(end)+2) = NewResistanceLevel; %new

                  end

            end

      end

end

end

***************************

**(5) Mitigation_Scenario3_Neighbor_Mod.m**

 function [Parcel, Damage_year, Upgrade_year_tracker] = Mitigation_Scenario3_Neighbor_Mod(Parcel, ...

    Damage_year, threshold_Neigh,year, Upgrade_year_tracker, Neigh_List)

%This function is for mitigation Option 3 - after a storm, there is some likelihood

%that an undamaged house will increase its resistance level if some percentage of neighbors

%experience damage. This is run after case 2_Base.

%Rule: for a house the is undamaged, if the more than threshold_Neigh% of the

%      neighbors (i.e., houses within 75m) are damaged in 'year', they will

%      improve the construction quality one resistance level

%Called by: Hazard_SEES_ABM.m

%Calls: Returns_NewResistanceLevel.m

%Input:

%Upgrade_year_tracker: Counts the number of upgrades that are made each year

%***************************

%Find the list of active parcels that are neighbors with a damaged house but

%are themselves not damaged

%***************************

%find the neighbors (ID) of damaged houses who themselves are not damaged

ParcelIDs_damaged = Damage_year(Damage_year(:,7)>1,1);

NeighborToDamagedParcel = zeros(size(Damage_year,1),1);

next=1;

for i = 1:size(ParcelIDs_damaged,1) %add neighbors of ParcelIDs_damaged(i) to list if they are not

damaged

    Neigh=Neigh_List(ParcelIDs_damaged(i),Neigh_List(ParcelIDs_damaged(i),:)>0);

    for j = 1:size(Neigh,2)

        if ~ismember(Neigh(j),ParcelIDs_damaged) && ~ismember(Neigh(j),NeighborToDamagedParcel)

%is neigh(i) is damaged, if yes, don't add to list

            NeighborToDamagedParcel(next,1) = Neigh(j);

            next=next+1;

        end

    end

end

NeighborToDamagedParcel = NeighborToDamagedParcel(NeighborToDamagedParcel(:,1)>0,1);

for i =1:size(NeighborToDamagedParcel,1)

    %determine how many neighbors to i that are damaged

    Neigh = Neigh_List(NeighborToDamagedParcel(i),Neigh_List(NeighborToDamagedParcel(i),:)>0);

    NumNeigh = size(Neigh,2);

    NumDam2 = 0;

    %find Neigh that are damaged

    for j = 1:size(Neigh,2)

        if Damage_year(Neigh(j),7)>1

          NumDam2=NumDam2+1;

        end

    end

    BuildingType = Parcel(NeighborToDamagedParcel(i),3);

    ResisLevel_current = Parcel(NeighborToDamagedParcel(i),4);

    if NumDam2/NumNeigh > threshold_Neigh %0.5

       temp = Returns_NewResistanceLevel(ResisLevel_current,BuildingType);

       Parcel(NeighborToDamagedParcel(i),4) = temp;

       Damage_year(NeighborToDamagedParcel(i),7) = temp;

       temp2 = find(Upgrade_year_tracker(NeighborToDamagedParcel(i,1),:)>0);

       if temp2(end)<10

           Upgrade_year_tracker(NeighborToDamagedParcel(i,1),temp2(end)+1) = year;

           Upgrade_year_tracker(NeighborToDamagedParcel(i,1),temp2(end)+2) = temp;

       end

    end

end

end

***************************

**(6) Mitigation_Scenario4_Policy.m**

function [Parcel,Upgrade_year_tracker] = Mitigation_Scenario4_Policy(Parcel, percentHouseGivenSubsidy, year,Upgrade_year_tracker)

% This function is for mitigation Option 4 - some percentage of homes (regardless of damage) are given % subsidies to upgrade. Subsidies are not given to houses that are in resistance level 7 (max).

%Called by: Hazard_SEES_ABM.m

%Parcels that are active and have at resistance level of 6 or less. A double.

Parcels_Active=Parcel(isnan(Parcel(:,2)),:); %active parcels

Parcel_subset_IDs_NaN7 = Parcels_Active(Parcels_Active(:,4)<Parcels_Active(:,6),1);

%Number of active parcels, parcel given subsidy, number of parcels eligble for subsidy

NumParcels = size(Parcels_Active,1); %number of active parcels; includes houses with RL 7

NumParcelsGivenSubsidy =round(percentHouseGivenSubsidy*NumParcels);

NumParcels_NaN7 = size(Parcel_subset_IDs_NaN7,1); %num active parcels eligible for the subsidy

if NumParcels_NaN7<NumParcelsGivenSubsidy   %number of houses that are active and with RL less

      %     than 7 is fewer than the x% choosen by the government for an upgrade.

      NumParcelsGivenSubsidy=NumParcels_NaN7;

elseif NumParcels_NaN7==0  %number of houses that are active and with RL less than 7 is 0

      %ParcelIDs_GivenSubsidy=NaN;

      return; %No parcel is eligible for a subsidy

end

ParcelIDs_GivenSubsidy = Parcel_subset_IDs_NaN7(randperm(NumParcels_NaN7,NumParcelsGivenSubsidy),1);

      %randomly choose NumParcelsGivenSubsidy in NumParcels_NaN7 list for an upgrade

for i = 1:NumParcelsGivenSubsidy

      ResisLevel_current = Parcel(ParcelIDs_GivenSubsidy(i,1),4);

      BuildingType = Parcel(ParcelIDs_GivenSubsidy(i,1),3);

      temp= Returns_NewResistanceLevel(ResisLevel_current,BuildingType);

      Parcel(ParcelIDs_GivenSubsidy(i,1),4) =temp;

      temp2 = find(Upgrade_year_tracker(ParcelIDs_GivenSubsidy(i,1),:)>0);

      if temp2(end) < 10

          Upgrade_year_tracker(ParcelIDs_GivenSubsidy(i,1),temp2(end)+1) = year;

          Upgrade_year_tracker(ParcelIDs_GivenSubsidy(i,1),temp2(end)+2) = temp;

      end

end

end

***************************

**(7) Returns_NewResistanceLevel.m**

function NewResistanceLevel =  Returns_NewResistanceLevel(ResistanceLevel, BuildingType)

%Called by  Mitigation_Option3.m

%           Mitigation_Option4.m

if BuildingType < 6

    if ResistanceLevel==1

        NewResistanceLevel = 2;

    elseif  ResistanceLevel==2

        y = rand();

        if y>0.5

            NewResistanceLevel = 3;

        else

            NewResistanceLevel = 4;

        end

    elseif ResistanceLevel==3

        NewResistanceLevel = 5;

    elseif  ResistanceLevel==4

        NewResistanceLevel = 5;

    elseif  ResistanceLevel==5

        NewResistanceLevel = 6;

    else

        NewResistanceLevel = 6;

    end

elseif BuildingType < 10

    if ResistanceLevel==1

        NewResistanceLevel = 2;

    elseif  ResistanceLevel==2

        y = rand();

        if y>0.5

            NewResistanceLevel = 3;

        else

            NewResistanceLevel = 4;

        end

    elseif  ResistanceLevel==3

        NewResistanceLevel = 5;

    elseif  ResistanceLevel==4

        NewResistanceLevel = 6;

    elseif  ResistanceLevel==6

        NewResistanceLevel = 7;

    elseif  ResistanceLevel==5

        NewResistanceLevel = 7;

    else

        NewResistanceLevel=7;

    end

end

if BuildingType == 10

    if ResistanceLevel==1

        NewResistanceLevel = 2;

    else

        NewResistanceLevel = 2;

    end

end

if BuildingType == 11

    if ResistanceLevel==1

        NewResistanceLevel = 2;

    else

        NewResistanceLevel = 3;

    end

end

end

***************************

**(8) CountNumUpgrades_inReplication.m**

function NoUpgradesMaster = CountNumUpgrades_inReplication(Parcel, Parcel_Input, Parcel_forUpgadeCount)

numParcelsInitial = size(Parcel,1);

temp = Parcel(:,4)-Parcel_Input(:,4);

for index_Upgrade = 1:numParcelsInitial

    if Parcel_Input(index_Upgrade,3) < 6 && temp(index_Upgrade,1)>1

        if temp(index_Upgrade,1)>=2 && Parcel_forUpgadeCount(index_Upgrade,4)==2

            temp(index_Upgrade,1) = temp(index_Upgrade,1) - 1;

        elseif temp(index_Upgrade,1)>=2 && Parcel_forUpgadeCount(index_Upgrade,4)==3

            temp(index_Upgrade,1) = temp(index_Upgrade,1) - 1;

        end

    elseif Parcel_Input(index_Upgrade,3) < 10 && temp(index_Upgrade,1)>1

        if temp(index_Upgrade,1)==2 && Parcel_forUpgadeCount(index_Upgrade,4)==2

            temp(index_Upgrade,1) = temp(index_Upgrade,1) - 1;

        elseif temp(index_Upgrade,1)>2 && Parcel_forUpgadeCount(index_Upgrade,4)==2

            temp(index_Upgrade,1) = temp(index_Upgrade,1) - 2;

        elseif temp(index_Upgrade,1)==2 && Parcel_forUpgadeCount(index_Upgrade,4)==3

            temp(index_Upgrade,1) = temp(index_Upgrade,1) - 1;

        elseif temp(index_Upgrade,1)>2 && Parcel_forUpgadeCount(index_Upgrade,4)==3

            temp(index_Upgrade,1) = temp(index_Upgrade,1) - 2;

        end

    end

end

NoUpgradesMaster = temp;

end

***************************

**(9) save_data.m**

function save_data(replication, city, caseNo, Damage_Master_Year, Upgrade_year_tracker_Rep, ...

    NoUpgradesMaster, Upgrade_Sensitivity_factor, threshold_Neigh, percentHouseGivenSubsidy)

if caseNo==1

    X = strcat('Damage_Master_',city,'_Baseline_',int2str(replication),'.mat');

    parsave_Damage(X, Damage_Master_Year);

    Y = strcat('NoUpgradesMaster_',city,'_Baseline_',int2str(replication),'.mat');

    parsave_UpgradeNo(Y,NoUpgradesMaster,Upgrade_year_tracker_Rep);

elseif caseNo==3 && Upgrade_Sensitivity_factor >= 0

    X = strcat('Damage_Master_',city,'_NeighborCase_USensF',int2str(Upgrade_Sensitivity_factor*100),...

'_NeighT',int2str(threshold_Neigh*100),'_',int2str(replication),'.mat');

    parsave_Damage(X, Damage_Master_Year);

    Y=strcat('NoUpgradesMaster_',city,'_NeighborCase_USens',int2str(Upgrade_Sensitivity_factor*100),...

'_NeighT',int2str(threshold_Neigh*100),'_',int2str(replication),'.mat');

    parsave_UpgradeNo(Y,NoUpgradesMaster,Upgrade_year_tracker_Rep);

elseif caseNo==3 && Upgrade_Sensitivity_factor < 0

    X =strcat('Damage_Master_',city,'_NeighborCase_USensFm',int2str(Upgrade_Sensitivity_factor*100),...

'_NeighT', int2str(threshold_Neigh*100),'_',int2str(replication),'.mat');

parsave_Damage(X, Damage_Master_Year);

    Y = strcat('NoUpgradesMaster_',city,'_NeighborCase_USensFm',...

int2str(Upgrade_Sensitivity_factor*100),'_NeighT',int2str(threshold_Neigh*100),...

'_',int2str(replication),'.mat');

    parsave_UpgradeNo(Y,NoUpgradesMaster,Upgrade_year_tracker_Rep);

elseif caseNo==2 && Upgrade_Sensitivity_factor >= 0

    X = strcat('Damage_Master_',city,'_UpgradeCase_',int2str(Upgrade_Sensitivity_factor*100),...

'_',int2str(replication),'.mat');

    parsave_Damage(X, Damage_Master_Year);

    Y = strcat('NoUpgradesMaster_',city,'_UpgradeCase_',int2str(Upgrade_Sensitivity_factor*100),...

'_',int2str(replication),'.mat');

    parsave_UpgradeNo(Y,NoUpgradesMaster,Upgrade_year_tracker_Rep);

elseif caseNo==2 && Upgrade_Sensitivity_factor < 0

    X = strcat('Damage_Master_',city,'_UpgradeCase_m',int2str(Upgrade_Sensitivity_factor*100),...

'_',int2str(replication),'.mat');

    parsave_Damage(X, Damage_Master_Year);

    Y = strcat('NoUpgradesMaster_',city,'_UpgradeCase_m',int2str(Upgrade_Sensitivity_factor*100),...

'_',int2str(replication),'.mat');

    parsave_UpgradeNo(Y,NoUpgradesMaster,Upgrade_year_tracker_Rep);

elseif caseNo ==4

    X = strcat('Damage_Master_',city,'_PolicyCase_',int2str(percentHouseGivenSubsidy*100),...

'_',int2str(replication),'.mat');

    parsave_Damage(X, Damage_Master_Year);

    Y = strcat('NoUpgradesMaster_',city,'_PolicyCase_',int2str(percentHouseGivenSubsidy*100),...

'_',int2str(replication),'.mat');

    parsave_UpgradeNo(Y,NoUpgradesMaster,Upgrade_year_tracker_Rep);

end

end
